# The tumor-enriched small molecule gambogic amide suppresses glioma by targeting WDR1-dependent cytoskeleton remodeling

**DOI:** 10.1038/s41392-023-01666-3

**Published:** 2023-11-08

**Authors:** Jiaorong Qu, Bojun Qiu, Yuxin Zhang, Yan Hu, Zhixing Wang, Zhiang Guan, Yiming Qin, Tongtong Sui, Fan Wu, Boyang Li, Wei Han, Xiaozhong Peng

**Affiliations:** 1 Department of Molecular Biology and Biochemistry, Institute of Basic Medical Sciences, Medical Primate Research Center, Neuroscience Center, Chinese Academy of Medical Sciences, School of Basic Medicine, Peking Union Medical College, Beijing, 100005 China; 2 State Key Laboratory of Common Mechanism Research for Major Diseases, Beijing, China; 3 Department of Molecular Neuropathology, Beijing Neurosurgical Institute, Capital Medical University, Beijing, 100070 China; 4 Department of Neurosurgery, Beijing Tiantan Hospital, Capital Medical University, Beijing, China; 5 State Key Laboratory of Respiratory Health and Multimorbidity, Beijing, China; 6 National Human Diseases Animal Model Resource Center, Beijing Engineering Research Center for Experimental Animal Models of Human Critical Diseases, Institute of Laboratory Animal Science, Chinese Academy of Medical Sciences & Peking Union Medical College, Beijing, 100021 China

**Keywords:** CNS cancer, Drug development, Target identification, Cancer stem cells, Drug development

## Abstract

Glioma is the most prevalent brain tumor, presenting with limited treatment options, while patients with malignant glioma and glioblastoma (GBM) have poor prognoses. The physical obstacle to drug delivery imposed by the blood‒brain barrier (BBB) and glioma stem cells (GSCs), which are widely recognized as crucial elements contributing to the unsatisfactory clinical outcomes. In this study, we found a small molecule, gambogic amide (GA-amide), exhibited the ability to effectively penetrate the blood-brain barrier (BBB) and displayed a notable enrichment within the tumor region. Moreover, GA-amide exhibited significant efficacy in inhibiting tumor growth across various in vivo glioma models, encompassing transgenic and primary patient-derived xenograft (PDX) models. We further performed a genome-wide clustered regularly interspaced short palindromic repeats (CRISPR) knockout screen to determine the druggable target of GA-amide. By the combination of the cellular thermal shift assay (CETSA), the drug affinity responsive target stability (DARTS) approach, molecular docking simulation and surface plasmon resonance (SPR) analysis, WD repeat domain 1 (WDR1) was identified as the direct binding target of GA-amide. Through direct interaction with WDR1, GA-amide promoted the formation of a complex involving WDR1, MYH9 and Cofilin, which accelerate the depolymerization of F-actin to inhibit the invasion of patient-derived glioma cells (PDCs) and induce PDC apoptosis *via* the mitochondrial apoptotic pathway. In conclusion, our study not only identified GA-amide as an effective and safe agent for treating glioma but also shed light on the underlying mechanisms of GA-amide from the perspective of cytoskeletal homeostasis.

## Introduction

Glioma is one of the most common primary tumors of the central nervous system (CNS), accounting for 81% of malignant tumors in the CNS, and originates mainly from glial tissue.^1^ According to the new 2021 World Health Organization classification of CNS tumors, gliomas are classified into grades 1–4. Glioblastoma (GBM) is classified as the most malignant grade (grade 4),^2^ with a high incidence and mortality and a poor prognosis.^3^ The current standard of care is maximal safe surgical resection followed by radiotherapy and adjuvant chemotherapy with temozolomide (TMZ).^4^ The invasive growth pattern of GBM hinders the complete resection of all tumor tissues,^5^ making postoperative adjuvant therapies crucial for patient prognosis. However, it is concerning to note that approximately 60% of patients do not respond to postoperative TMZ treatment.^6^ As a result, tumor recurrence occurs in almost all patients with GBM.

One of the reasons for the poor chemotherapeutic outcomes in glioma is the existence of the blood‒brain barrier (BBB), the barrier between the circulation and brain tissue.^7^ The presence of the BBB hampers the entry of most macromolecular and small molecule drugs into the brain, thereby rendering novel inhibitors that have shown remarkable efficacy in other tumor types ineffective in GBM.^8^ The presence of glioma stem cells (GSCs) serves as an additional influential factor contributing to the limited efficacy of chemotherapy in the treatment of gliomas.^9,10^ GSCs are responsible for malignant features such as tumor heterogeneity, cellular hierarchy,^11^ high invasiveness,^12^ angiogenic activity,^13^ treatment resistance,^14,15^ and the formation of the blood-tumor barrier.^16^ Therefore, there is an urgent need to explore new compounds that can cross the BBB and eliminate glioma cells (GCs) and GSCs. In our early research, we applied an unbiased drug screen of 1920 compounds and identified a small molecule compound, gambogic amide (GA-amide), which could target GSCs.^17^ GA-amide, an analog of gambogic acid (GAC) that constitutes a primary active constituent of the traditional Chinese medicine, gamboge, was originally identified as a selective tropomyosin receptor kinase A (TrkA) agonist and nerve growth factor-mimetic small molecule.^18^ Specifically, GA-amide could activate TrkA by inducing its phosphorylation at Y490, Y751 and Y794.^19^

Previous studies have demonstrated the neuroprotective potential of GA-amide in vitro and in vivo,^18^ as well as its reported effects in promoting bone formation,^20^ preventing hair graying, and accelerating hair growth.^21^ Furthermore, GA-amide exhibited potential in cancer treatment by inhibiting leukemia cell proliferation and reducing leukemia progression in vivo;^22^ while also markedly increasing interleukin 6 (IL-6) secretion and the interferon gamma (IFN-γ) response in breast carcinoma.^23^ In our previous research, GA-amide was found to inhibit angiogenesis in a TrkA-independent manner,^24^ which indicating that GA-amide might hold considerable potential as an inhibitor of cancer progression. Additionally, its neuroprotective properties rendered it advantageous for treating CNS diseases.

In this article, we investigated the inhibitory effect of GA-amide on patient-derived GCs (PDCs) and GSCs in vitro and in vivo. In previous studies, subcutaneous injection of 2 mg/kg GA-amide was found to significantly reduce the infarct volume in a transient middle cerebral artery occlusion stroke model,^18^ suggesting that it could cross the BBB. Here, we evaluated the BBB permeability and the safety of GA-amide in vivo. Moreover, we screened for the target of gambogic amide and identified it as WD repeat domain 1 (WDR1) rather than TrkA that was considered as its historically accepted target. Then, we studied the mechanism of gambogic amide and clarified that gambogic amide exerted its anti-glioma effect by binding to its direct functional target.

## Results

### GA-amide specifically inhibited GSCs and PDCs in vitro

**Fig. 1:**
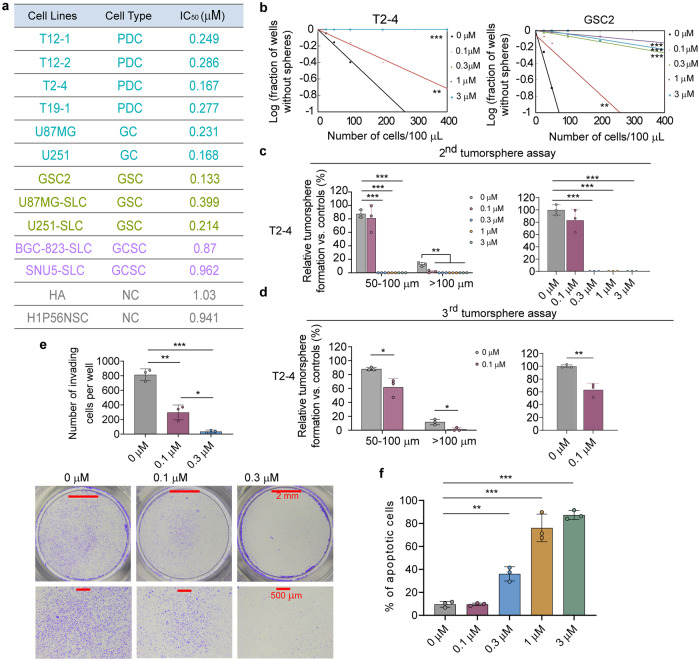
The antiglioma effect of GA-amide in vitro**. a** The IC_50_ values of GA-amide in four PDC, two GC, three GSC, two GCSC and two normal human cell lines were determined by cell viability assays. **b** Limiting dilution assay of T2-4 and GSC2 cells after pretreatment with GA-amide for 4 h. For T2-4, the three lines that represent the treatment of 0.3 μM, 1 μM and 3 μM were overlapped. Data were analyzed by ELDA, *n* = 10 for each group. **c** Secondary tumor sphere assay of T2-4 cells after pretreatment with GA-amide for 4 h. **d** Tertiary tumor sphere assay of T2-4 cells collected from secondary tumor spheres that were pretreated with GA-amide for 4 h. **e** Transwell invasion assays were used to detect T2-4 cell invasion after GA-amide treatment for 48 h, and the number of invading cells per field was determined. The lower panel shows images of the Transwell assay. Scale bars: 2 mm, 500 μm. **f** Flow cytometry–based quantification of T2-4 cell apoptosis after 4 h of exposure to different concentrations of GA-amide by annexin V/PI staining. In (**c**–**f**), the data are presented as the mean ± SEMs, and the samples were assayed in triplicate. The data in (**c**–**f**) were analyzed by ANOVA, *P < 0.05, **P < 0.01, ***P < 0.001

Firstly, in order to assess the selectivity of GA-amide in 13 cell lines, following treatment with different concentrations of GA-amide, a cell viability assay was applied to determine the half-maximal inhibitory concentration (IC_50_) of GA-amide in the various cell lines. We found that GA-amide specifically reduced the viability of glioma-related cells, including PDCs, GCs and GSCs, while the IC_50_ values of GA-amide in other cancer stem cells (gastric cancer stem cells, GCSCs) and nontumor cells were much higher, indicating that glioma-related cells were more sensitive for GA-amide treatment (Fig. 1a). Moreover, as the self-renewal ability reflects the stemness characteristics of GCs, a limiting dilution assay was performed to evaluate the changes in stemness characteristics after GA-amide pretreatment for 4 h. The results revealed that self-renewal was suppressed in both PDCs (T2-4, T12-1, T12-2) and GSC2 cells (Fig. 1b and Supplementary Fig. 1a). To further assess the continuous effects of GA-amide on PDCs and GSCs, tumor sphere formation assays were used. PDCs were pretreated with GA-amide for 4 h and collected to assess the inhibition of tumor sphere formation by PDCs after GA-amide withdrawal. The results showed that the secondary tumor sphere formation potential was decreased; strikingly, it was completely abolished in PDCs after pretreatment with 0.3 μM, 1 μM and 3 μM GA-amide (Fig. 1c and Supplementary Fig. 1b). The viable cells from the secondary sphere formation assays were collected and dissociated for the tertiary tumor sphere formation assays. Tertiary tumor sphere formation derived form T2-4 and T12-2 was still suppressed, indicating that a single treatment of GA-amide exerted inhibitory effects for tumor formation even after a second passage (Fig. 1d and Supplementary Fig. 1c). Additionally, given the highly invasive nature of of GBM, we assessed the invasiveness of PDCs and GSCs by treatment with the compound at 0.1 μM and 0.3 μM for a duration of 48–72 h. In this experiment, to exclude interference from the inhibitory effect of GA-amide on cell proliferation, we increased the seeding density of PDCs in the wells by 10-fold compared with that used in the IC_50_ assay, since the proliferation of PDCs was not inhibited at this high density. As expected, our observations revealed a significant decrease in the number of invading PDCs and GSCs as demonstrated by the transwell assay (Fig. 1e and Supplementary Fig. 1d, e). Furthermore, PDCs were subjected to a 4 h treatment with varying concentrations of GA-amide, and subsequent collected to assess apoptosis. As shown in Fig. 1f and Supplementary Fig. 1f, GA-amide treatment significantly induced the apoptosis of PDCs in a dose-dependent manner. Collectively, these results demonstrated the pharmacological inhibitory effects of GA-amide on both PDCs and GSCs.

### GA-amide treatment was safe in vivo, exhibited BBB permeability and specifically targeted the tumor area

**Fig. 2:**
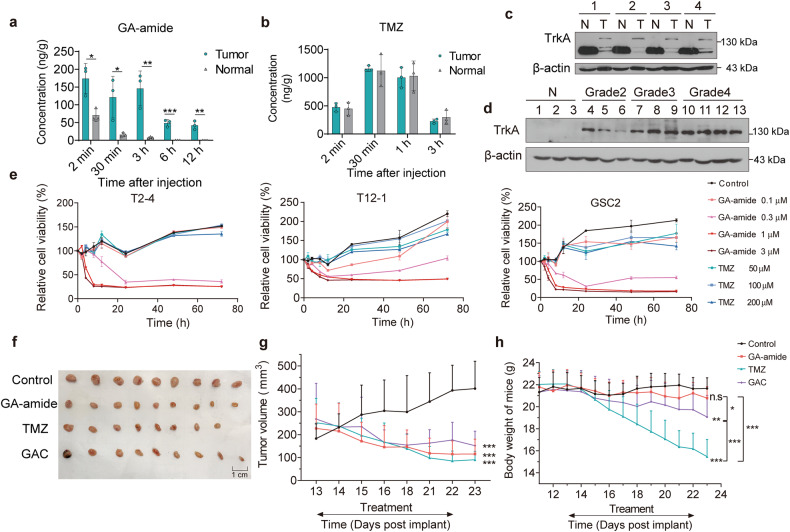
The BBB permeability and tumor suppressor ability of GA-amide compared with TMZ. GA-amide (**a**) or TMZ (**b**) concentration in the tumor area and normal brain tissue following a single injection of GA-amide (10 mg/kg, i.v.) or TMZ (10 mg/kg, i.v.). The data are presented as the means ± SEMs (*n* = 3). **c** Western blot analysis of the expression of TrkA in mouse tumor tissues (T) and normal brain tissues (N), *n* = 4. **d** Western blot analysis of glycosylated TrkA expression in human normal brain tissues (N) and glioma tissues of different grades. **e** Time curve of GA-amide and TMZ treatment in a cell viability assay (MTS) in T2-4, GSC2 and T12-1 cells. **f**–**h** A PDX mouse model was established, and the mice were treated with vehicle (control, *n* = 9), 2 mg/kg GA-amide (*n* = 9), 2 mg/kg GAC (*n* = 9) or 60 mg/kg TMZ (*n* = 8) by i.p. injection daily for 10 days. A summary photograph is presented in (**f**), statistical results in (**g**) and the body weights of mice in (**h**); the mean ± SEMs are shown. n.s: *P* ≥ 0.05, *P < 0.05, **P < 0.01 and ***P < 0.001 compared with the control group by one-way ANOVA at each time point

TMZ is an orally administered alkylating agent with BBB permeability and currently regarded as the standard therapeutic agent for the treatment of newly diagnosed GBM.^25,26^ Here, a U87MG-SLC-derived intracranial xenograft mouse model was established to compare the BBB permeability of TMZ and GA-amide. Interestingly, the concentration of GA-amide reached its peak just 2 min after intravenous injection of GA-amide, with a measured value at 174 ng/g of tissue. Notably, the concentration in the tumor area was found to be as least twice as high as that in the nontumor area, indicating that GA-amide specifically targets the tumor area (Fig. 2a). Although TMZ was detectable in both the tumor area and nontumor area of the brain, there was no discernible difference in its distribution between these regions (Fig. 2b). As previously reported, GA-amide was found to bind with TrkA at the juxtamembrane regions, an event that was determined to be necessary for the membrane penetration of GA-amide since the deficiency of TrkA in the area prevented the entry of GA-amide into the cells.^18^ Based on this, we examined the expression of TrkA in normal brain tissues and glioma tissues from the same mice. Results showed that glycosylated TrkA, with a molecular weight of 140 kDa, which facilitates the membrane penetration of GA-amide, was especially abundant in mouse tumor tissues (Fig. 2c). Moreover, TrkA expression was evaluated in normal tissue and glioma tissues of different grades from humans. Remarkably, our analysis revealed that TrkA exhibited high levels of expression in glioma tissues, whereas it was barely detectable in normal brain tissues (Fig. 2d). This might explain the tumor-specific enrichment of GA-amide and indicate that patients with high TrkA expression might be more sensitive to GA-amide due to the facilitating effect of TrkA on the cell membrane penetration of GA-amide. Furthermore, continuous monitoring of cell viability revealed that GA-amide effectively reduced the viability of TMZ-resistant cells, including PDCs and GSCs (Fig. 2e). Moreover, subcutaneous PDX models of glioma were established to further determine the in vivo anticancer activity of GA-amide compared with TMZ and GAC, an analog of GA-amide. We divided tumor-bearing mice into four treatment groups: control (vehicle), GA-amide, TMZ and GAC and observed that the tumor volumes in the GA-amide, GAC and TMZ treatment groups were significantly decreased compared with those in the control group (Fig. 2f, g). Immunohistological analysis of representative tumor sections from the control and GA-amide-treated tumors at the endpoint showed decreased expression of Ki67 and the GSC marker prominin 1 (CD133) as well as an increased level of cleaved caspase3 in GA-amide-treated tumors (Supplementary Fig. 2), suggesting that GA-amide inhibited glioma in vivo. Moreover, the body weight of mice treated with TMZ significantly decreased, but GA-amide did not cause the loss of body weight (Fig. 2h). Besides, following treatment with GA-amide, the liver, kidney and small intestine of mice revealed no discernible abnormalities (Supplementary Fig. 3a). Additionally, in the acute toxicity assay, GA-amide did not change the body weight of mice after a single injection (low dose: 1 mg/kg, high dose: 10 mg/kg) (Supplementary Fig. 3b). Moreover, there were no significant differences in routine blood parameters and most analytes evaluated in biochemical analysis of mouse serum; however, the levels of alanine aminotransferase (ALT) and Cl fluctuated (Supplementary Tables 1, 2). These results strongly indicated that GA-amide is a safe and well-tolerated small molecule for the treatment of glioma.

### GA-amide inhibited the growth of glioma in vivo in xenograft models and transgenic mouse models

**Fig. 3:**
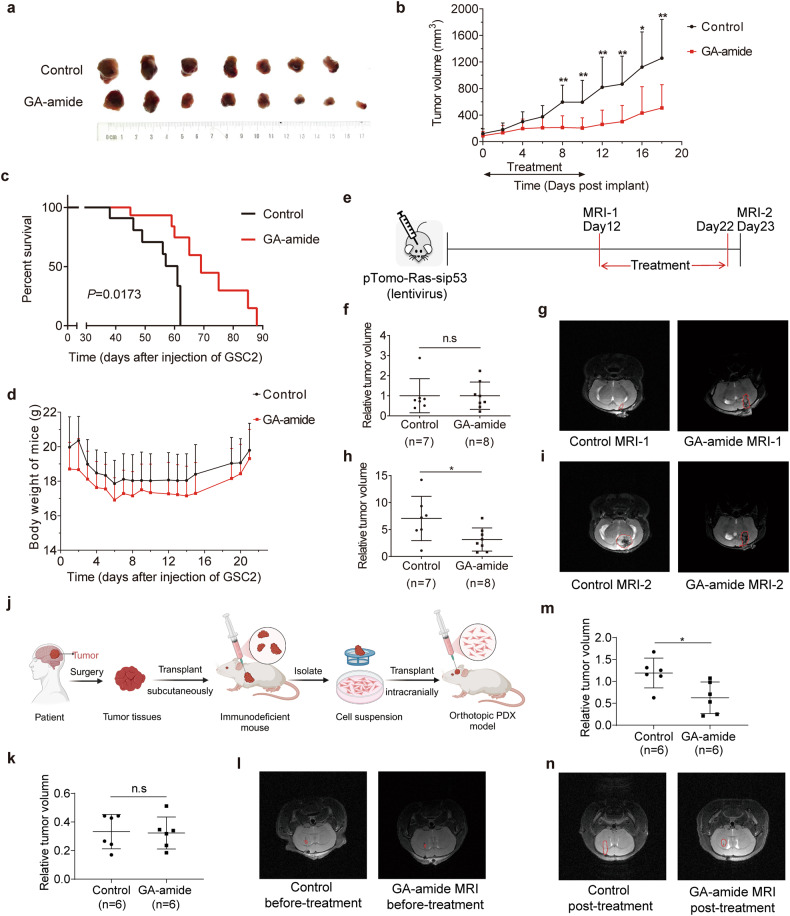
The inhibitory effects of GA-amide in glioma stem cell-derived mouse models and transgenic mouse models. **a**, **b** GSC2 cells were implanted s.c. into nude mice. Mice were treated with control (DMSO) (*n* = 7) or GA-amide (2 mg/kg) (*n* = 8) *via* i.p. injection once daily for 11 days, after which a photograph (**a**) was taken to show the tumor volume, and the tumor volume was calculated by a modified ellipsoid formula in (**b**). **c**, **d** GSC2 cells were implanted intracranially into nude mice. Mice were injected i.v. with control (*n* = 8) or 1 mg/kg GA-amide (*n* = 8) daily for 13 days, the Mantel‒Cox log–rank test was used for analysis, and the relative survival curves are shown in (**c**). The body weights are shown in (**d**). **e** In vivo efficacy studies of GA-amide using transgenic mouse models. Glioma was induced in transgenic C57BL/6 mice by injection of pTomo-Ras-sip53 lentivirus, and the strategy is shown. **f**, **g** MRI analysis was used to group mice into the control (*n* = 7) and GA-amide-treated groups (*n* = 8) before treatment. The statistical results are shown in (**f**), and representative MRI images are shown in (**g**). Mice were treated with vehicle (control) or 1 mg/kg GA-amide i.v. daily for 11 days, and the drug effects (**h**) and representative MRI images (**i**) are shown. **j** The establish of the intracranial orthotopic PDX model. **k**, **l** MRI analysis was used to group mice into the control (*n* = 6) and GA-amide-treated groups (*n* = 6) before treatment. The statistical results are shown in (**k**), and representative MRI images are shown in (**l**). Mice were treated with vehicle (control) or 1 mg/kg GA-amide i.v. daily for 10 days, and the drug effects (**m**) and representative MRI images (**n**) are shown. In (**b**, **d**, **f**, **h**, **k** and **m**), the data are presented as the mean ± SEMs. n.s: *P* ≥ 0.05, *P < 0.05, **P < 0.01, ***P < 0.001 compared with the control group by 2-tailed Student’s *t* test

To further clarify the effect of GA-amide in vivo, we applied GSC-derived subcutaneous xenograft models, wherein GA-amide were administered i.p.at a dose of 2 mg/kg over 11 days and found that the administration of GA-amide significantly inhibited the growth of tumors (Fig. 3a, b). To further investigate the therapeutic effects of GA-amide on intracranial xenografts models, we treated nude mice bearing GSC2-derived intracranial xenografts with GA-amide (1 mg/kg, i.v.) daily for 13 days. Nude mice treated with GA-amide exhibited prolonged survival times (Fig. 3c), but showed no significant difference in mouse body weight between the treatment group and control group (Fig. 3d). Additionally, a transgenic mouse model was applied as a primary brain tumor model to simulate the characteristics of primary gliomas. Specifically, the brains of mice in this model were injected with lentivirus to activate H-RasV12 and silence p53, which was shown in Fig. 3e along with the treatment strategy. Twelve days after injection, the mice were grouped into the GA-amide treatment and control groups depending on the magnetic resonance imaging (MRI) results, with the aim of ensuring that there were no differences between the control group and the GA-amide-treated group (Fig. 3f, g). Following administration of GA-amide or vehicle, we performed a subsequent MRI was performed to assess the inhibitory effect of 1 mg/kg GA-amide treatment and found that the tumor size in the GA-amide-treated mice was significantly smaller compared to the control mice (Fig. 3h, i). Immunohistochemical staining of tumor sections from the GA-amide-treated transgenic mice revealed lower expression of the proliferation marker Ki67 (Supplementary Fig. 4a) and GSC marker CD133 (Supplementary Fig. 4b). Furthermore, we utilized intracranial orthotopic PDX model (Fig. 3j) as previously reported^27^ to further evaluated the in vivo efficacy of GA-amide. Seven days after injection, the mice were divided into two groups without significant difference in tumor volume based on the results from MRI (Fig. 3k, l). After 10 days of GA-amide (1 mg/kg, i.v.) treatment, a subsequent MRI was conducted to assess the inhibitory efficacy and showed that the tumor volume in the GA-amide-treated mice was significantly smaller compared to the control mice (Fig. 3m, n).

### Genome-wide clustered regularly interspaced short palindromic repeats (CRISPR) screens identified GA-amide-sensitive genes in PDCs

**Fig. 4:**
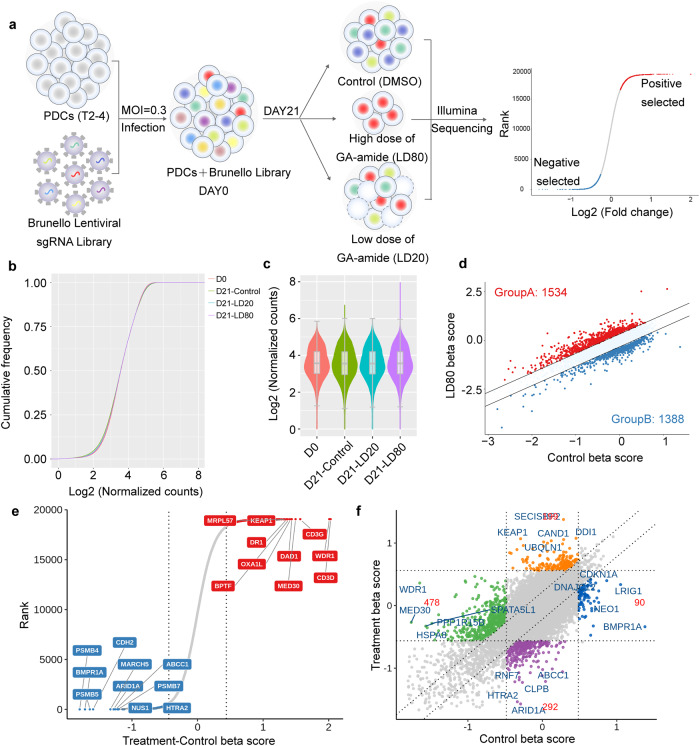
The genome-wide CRISPR/Cas9 screening for drug sensitive genes**. a** Schematic diagram illustrating the workflow of genome-wide CRISPR/Cas9 knockout library screening. The sgRNA cumulative frequency distribution (**b**) and the sgRNA distribution box (**c**) were used to preliminarily judge the variation of sgRNA in D0-DMSO, D21-DMSO, D21-LD20, and D21-LD80. **d** MAGeCKFlute was used to analyze the difference of normalized beta score between treatment and control (Δbeta-score) to identify positive selected genes (Group A in red) and negative selected genes (Group B in blue) in High-dose group (LD80). **e** Rank order plot depicting differential beta-scores between treatment and control. Top 10 hits of positive selection and negative selection were shown. **f** The nine-quadrant diagram showed sgRNA sequencing results of GA-amide treatment for 21 days

Although we have proved the efficacy and safety of GA-amide both in vitro and in vivo, the underlying mechanisms of GA-amide have not been adequately elucidated. It has been reported that GA-amide is a TrkA agonist, which can induce TrkA phosphorylation.^18,19^ Consistent with this report, we found that TrkA was phosphorylated at Y751 and Y490 in GSCs (Supplementary Fig. 5a, b); thus, we sought to determine whether the activity of TrkA is related to the inhibition of glioma. However, treatment with neither the TrkA inhibitor GW441756 nor the Trk family inhibitor K252a^28^ restored the inhibitory effect of GA-amide on GSCs, indicating that TrkA is not a functional target for GA-amide (Supplementary Fig. 5c, d). Therefore, to identify the druggable target of GA-amide, we performed a genome-scale CRISPR screen with the highly optimized Brunello CRISPR single-guide RNA (sgRNA) library, which contained a total of 76,441 guides with 4 guides per gene.^29,30^ The flowchart of the screening procedure is shown in Fig. 4a. Briefly, cells (1.2 × 10^8^) was transduced with the lentiviral library at a multiplicity of infection (MOI) of 0.3 to achieve 300 × coverage of each sgRNA, and then maintained in the presence of 1 μg/mL puromycin for 72 h to allow selection and gene editing. T2-4 cells successfully transduced with the lentiviral sgRNA library were treated with a high dose (LD80) of GA-amide, a low dose (LD20) of GA-amide or dimethyl sulfoxide (DMSO) for 21 days and then performed for the deep sequencing and related analyses to discovery the distribution of sgRNAs. As a result, approximately 99.9% of the sgRNA sequences were retained in all samples, which ensured nearly complete library coverage for the CRISPR library screen (Fig. 4b). Meanwhile, the sgRNA distribution in the GA-amide-treated groups on Day 21 was significantly different from the sgRNA distribution in the DMSO-treated cells (Fig. 4c). By analyzing the sgRNAs in the control and high-dose GA-amide-treated groups on Day 21, we identified 1534 positively enriched genes that functioned as drug-sensitive genes (Fig. 4d, *Data file S1*); the top 10 genes are shown in Fig. 4e. The nine-quadrant diagram displayed the top 5 positively selected genes that were distinct from the essential gene (Fig. 4f). Analysis of the results showed that there were 601 positively selected genes (*P* < 0.05) overlapping between the LD20- and LD80-GA-amide-treated groups compared with the DMSO-treated group (Supplementary Fig. 6a). Through Gene Ontology (GO) enrichment analysis of the 601 overlapping genes, we found that numerous enriched genes were associated with mitochondria and apoptotic processes (Supplementary Fig. 6b). In Kyoto Encyclopedia of Genes and Genomes (KEGG) pathway analysis, the overlapping positively selected genes were enriched in the apoptosis pathway (Supplementary Fig. 6c). Moreover, gene set enrichment analysis (GSEA) showed that apoptosis was significantly affected (Supplementary Fig. 6d). Along with the early results indicating that PDC apoptosis was induced by GA-amide, we also found that these drug-sensitive genes were enriched in the apoptosis pathway, which was possibly mitochondria-mediated.

### WDR1 was a druggable target of GA-amide that could restore the inhibitory effects of GA-amide on PDCs

**Fig. 5:**
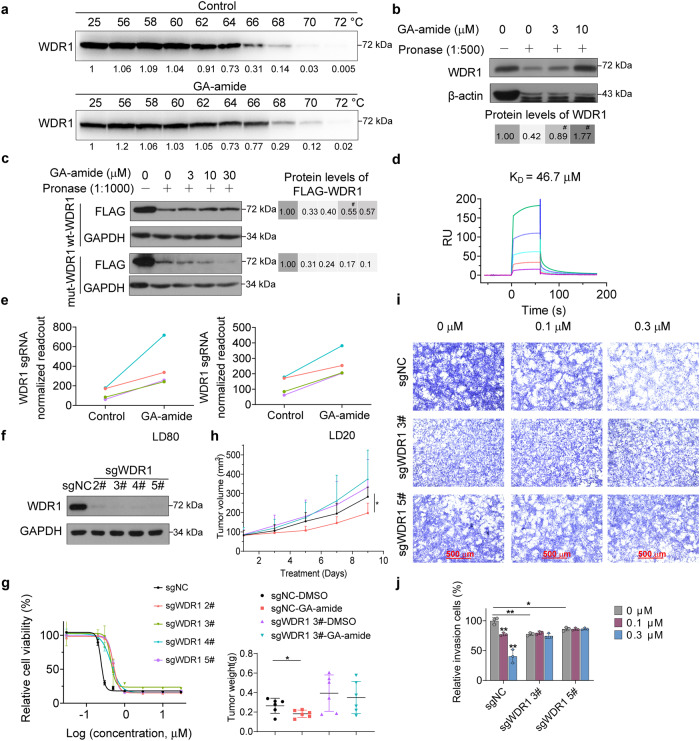
WDR1 is the direct target of GA-amide. **a** Western blot analysis showed that GA-amide protected WDR1 at different temperature gradients in the CETSA in T2-4 cells. **b** Western blot analysis showed that GA-amide promoted the resistance of WDR1 to pronase digestion in a dose-dependent manner in the DARTS assay in T2-4 cells. **c** Western blot analysis showed that GA-amide promoted the resistance of wt-WDR1 but not mut-WDR1 to pronase in a dose-dependent manner in the DARTS assay in 293T cells. **d** The direct interaction between GA-amide and WD2-7 (including the four predicted binding sites) was confirmed by SPR analysis. **e** The enrichment of four sgRNAs targeting *WDR1* in CRISPR/Cas9 screening. **f** The effect of sgRNAs on *WDR1* knockout in T2-4 cells. **g** Knockout of *WDR1* by any of the four sgRNAs prevented the inhibition of cell viability after GA-amide treatment, and samples were assayed in triplicate. **h** The sgNC and sg*WDR1* 3# U87MG-SLC cells were implanted s.c. into nude mice, which was treated with DMSO or GA-amide (2 mg/kg) *via* i.p. injection once daily for 10 days (*n* = 6). Then the tumor volume was calculated by a modified ellipsoid formula in the upper and the tumor weights were shown in the lower panel. **i** Transwell assays showed that *WDR1* knockout prevented the inhibition of invasion by GA-amide in T2-4 cells. The data are representative of 3 wells, scale bar: 500 μm. **j** Statistical results of the recovery of invasion. Mean ± SEM. *P < 0.05, **P < 0.01 and ***P < 0.001 compared with control by one-way ANOVA at each time point

We believed that the direct target of GA-amide was among the drug-sensitive genes; thus, we selected 14 genes—the top 10 positive drug-sensitive genes and 4 genes in the nine-quadrant diagram—as candidate target genes and sorted these genes in order of the Δbeta-score. Knockout of the compound’s target resulted in the loss of its binding to the target, thereby impeding its tumor-suppressive function. To verify drug targets among the candidate genes that interact with GA-amide, we applied a cellular thermal shift assay (CETSA), which is based on the principle that the binding of a ligand to its protein target protects the target from degradation at the permissive temperature.^31^ Western blot analysis of 11 candidate targets (those with an available antibody) showed that the band corresponding to WDR1 almost completely disappeared in the cells treated with DMSO at 67°C–70°C, while the band persisted in the cells treated with GA-amide (Fig. 5a and Supplementary Fig. 7a). Subsequently, we used another target identification technology, the drug affinity responsive target stability (DARTS) method^17,32^ to further investigate the engagement of the drug with its target. We found that GA-amide also significantly promoted WDR1 stabilization during pronase-induced degradation (Supplementary Fig. 7b). Moreover, as the concentration of gambogic amide incubated with the total protein of PDCs increased, the stability of WDR1 increased, indicating that GA-amide can bind to WDR1 (Fig. 5b). To further investigate the specific interaction sites of WDR1 with GA-amide, we performed molecular docking simulation using AutoDock.^31,33^ The simulated docking model of GA-amide and WDR1 as well as the four predicted binding sites (Lys-65, Asp-153, Arg-196 and Gln-288) were shown in Supplementary Fig. 7c, d. To verify this prediction, we build mut-WDR1 plasmid with four binding sites mutant and applied the DARTS method, finding that GA-amide protected wt-WDR1 but not mut-WDR1 (with mutation of the four interaction sites shown in Supplementary Fig. 7c) in 293T cells (Fig. 5c). In order to further investigate which specific site plays a crucial role, we constructed single residue mutant WDR1 plasmids and employed the DARTS method. Our findings revealed that the protection of GA-amide was decreased in the R196A-WDR1 mutant while other mutants at different residues still exhibited protection by GA-amide (Supplementary Fig. 7e). Hence, we speculated that while R196 may play a significant role in binding to GA-amide, the involvement of the other three residues cannot be overlooked as they might collectively participate in the binding process. Then, a third target identification technology, surface plasmon resonance (SPR) analysis,^34^ was performed to quantify the direct interaction of GA-amide with WDR1. We purified the His-WD2-7 protein containing the WD2 to WD7 repeats, which included the four interaction sites (Supplementary Fig. 7f) and applied for SPR analysis. The results exhibited a robust binding interaction between GA-amide and his-WD2-7, as evidenced by a *K*_*D*_ of 46.7 μM (Fig. 5d). In the results of the CRISPR/Cas9 library screen, all *WDR1*-targeting sgRNAs were dramatically enriched in high-dose GA-amide-treated T2-4 cells, implying that loss of *WDR1* caused the resistance of T2-4 cells to GA-amide treatment (Fig. 5e). To validate the results of screening, we established *WDR1* knockout PDCs by lentiviral transduction of sgRNAs included in the library and a control sgRNA (Fig. 5f and Supplementary Fig. 8a). *WDR1* knockout T2-4 and T12-1 cells showed decreased suppressive effects of GA-amide on cell proliferation (Fig. 5g and Supplementary Fig. 8b) in vitro. Furthermore, the in vivo experiment also showed that GA-amide could not inhibit the tumor growth of mice injected with *WDR1* deficient U87MG-SLC cells (Fig. 5h and Supplementary Fig. 8c, d). Moreover, 0.1 μM and 0.3 μM GA-amide treatment significantly inhibited the invasion of control sg-NC-transduced PDCs (T2-4 and T12-1 cells), but the inhibition of invasion by GA-amide was almost completely abolished in *WDR1* knockout PDCs (Fig. 5i, j and Supplementary Fig. 8e). Interestingly, we also observed that *WDR1*-deficient PDCs showed an attenuated invasion ability, consistent with a previous study showing that high WDR1 expression in GBM is positively correlated with poor prognosis.^35^ Based on the obvious effect of *WDR1* knockout on resistance to GA-amide treatment, we built WDR1 overexpressed cell line to investigate whether the overexpression of WDR1 could enhance the sensitivity of PDCs to GA-amide (Supplementary Fig. 9a). Although overexpressing WDR1 promoted PDC’s proliferation, it did not affect the effect of GA-amide on inhibiting cell proliferation (Supplementary Fig. 9b, c).

### GA-amide interacted with WDR1 to inhibit PDC invasion by disrupting cytoskeletal homeostasis

**Fig. 6:**
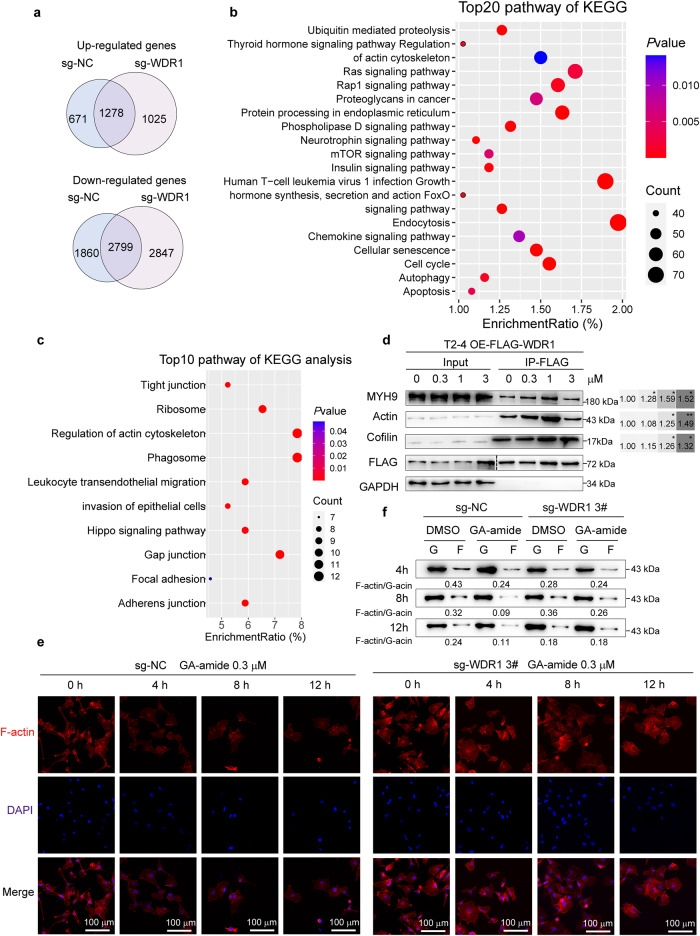
The combination of GA-amide and WDR1 disrupted the cytoskeleton to inhibit cell invasion. **a** The left circles represent the upregulated/downregulated genes in sg-NC T2-4 cells after 0.3 μM GA-amide treatment for 8 h; the right circles represent the upregulated/downregulated genes in sg-*WDR1* T2-4 cells. **b** KEGG enrichment analysis of the differentially expressed genes after GA-amide treatment identified by RNA-seq that were recovered in sg-*WDR1* T2-4 cells. **c** KEGG enrichment analysis of WDR1-interacting proteins after 3 μM GA-amide treatment for 1 h, as identified by IP-MS. **d** Western blot showing the expression of cytoskeleton-related proteins interacting with WDR1 after treatment with different concentrations of GA-amide in T2-4 cells. **e** TRITC-phalloidin staining of F-actin (red), DAPI staining of DNA (blue), and merged images in sg-NC and sg-*WDR1* T2-4 cells after 0.3 μM GA-amide treatment for 0, 4, 8 and 12 h, scale bar: 100 μm. **f** F-actin and G-actin extracted from sg-NC and sg-*WDR1* T2-4 cells treated with DMSO or GA-amide for 4, 8, and 12 h were subjected to immunoblotting with an anti-actin antibody

To address the mechanisms by which GA-amide suppresses the growth of PDCs, we performed gene expression profiling using RNA sequencing (RNA-seq). There were 1949 genes significantly upregulated and 4659 genes significantly downregulated in the sg-NC cell line after GA-amide treatment (*P* < 0.05). The upregulation of 1278 genes and downregulation of 2799 genes were recovered in the sg-*WDR1* cell line, and those genes were thus defined as recovered genes (Fig. 6a). To gain insight into the pathways regulated by GA-amide *via* WDR1, we investigated the recovered genes using KEGG analysis and found that the top 20 pathways included regulation of actin cytoskeleton and apoptosis (Fig. 6b). Given the previous reports indicating that WDR1 is a cytoskeleton-binding protein^36–38^ and considering the implications of the RNA-seq results, we suspected that GA-amide could inhibit glioma by affecting the binding of cytoskeleton-related proteins to WDR1. Thus, immunoprecipitation and mass spectrometry (IP-MS) experiments were further applied depending on T2-4 cells that overexpressed WDR1. We discovered that the differentially bound proteins were enriched in pathways such as regulation of actin cytoskeleton, migration, invasion, and cell junction after GA-amide treatment (Fig. 6c and Supplementary Fig. 10a). Consistent with analysis results of IP-MS, the in vitro IP experiment showed the interactions between WDR1 and cytoskeleton-related proteins such as myosin heavy chain 9 (MYH9), actin and Cofilin were enhanced (Fig. 6d and Supplementary Fig. 10b). These results suggested that GA-amide might inhibit glioma growth by modulating the cytoskeletal proteins that interact with WDR1; thus, we evaluated changes in the cytoskeleton and globular actin (G-actin)/microfilament (F-actin) expression after GA-amide treatment in T2-4 cells. After 4 h, 8 h and 12 h of treatment, the cells appeared flattened and collapsed, with a decreased content of F-actin at the cell edge (Supplementary Fig. 10c) accompanied by attenuated expression of F-actin (Supplementary Fig. 10d), which was responsible for the inhibition of cell invasion.^39^ Consistent with this finding, representative confocal images of F-actin visualized by TRITC-phalloidin staining showed that the F-actin content was decreased in sections from PDXs after treatment with GA-amide (Supplementary Fig. 10e). Furthermore, we applied jasplakinolide (jas), a stabilizer for the cellular cytoskeleton to co-retreated with GA-amide and found that once the F-actin was stabilized, the depolymerization of F-actin, the inhibition of cell invasion and the induction of apoptosis were significantly reversed (Supplementary Fig. 10f–h). To confirm the involvement of WDR1 in this process, we used *WDR1* knockout cells and found that the alteration in cytoskeleton (Fig. 6e) and the reduction in F-actin level that was observed in sg-NC cells with the treatment of GA-amide (Fig. 6f), were not observed in *WDR1* knockout cells. As we suggested that GA-amide promoted the combination of WDR1 with MYH9 and Cofilin, we established cell lines with knockdown of MYH9 or CFL1 in PDCs (Supplementary Fig. 11a) and conducted experiments to investigate whether the knockdown of MYH9 or CFL1 can reverse the effects of GA-amide. The results demonstrated that knocking down either MYH9 or CFL1 had significant reversing effects on GA-amide-induced destabilization of the cell cytoskeleton (Supplementary Fig. 11b, c) and inhibition of invasion (Supplementary Fig. 11d). Together, our results suggested that GA-amide directly interacted with WDR1 to form a complex with MYH9 and Cofilin, which worked together to accelerate the depolymerization of F-actin, resulting in the cytoskeletal remodeling and the ensuing inhibition of cell invasion.

### GA-amide induced glioma cell apoptosis *via* the mitochondrial apoptotic pathway

**Fig. 7:**
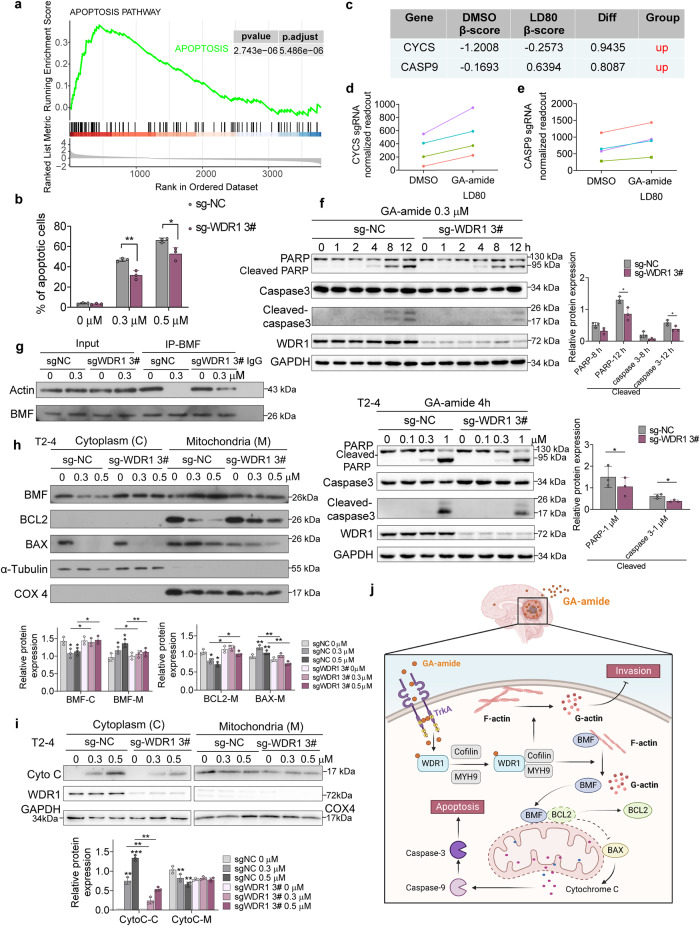
GA-amide induced PDC apoptosis *via* the WDR1-mediated mitochondrial apoptotic pathway**. a** GSEA of the apoptosis gene expression signature of the recovered genes identified by RNA-seq in sg-WDR1 T2-4 cells after GA-amide treatment. **b** Knockout of *WDR1* suppressed the induction of apoptosis after GA-amide treatment. **c**–**e** The CRISPR/Cas9 screening results of CYCS and CASP9 are shown in (**c**). The enrichment of four sgRNAs targeting CYCS (**d**) and CASP9 (**e**) in the screen. **f** Apoptosis-related markers were detected by western blotting in protein extracts from *WDR1* knockout and control T2-4 cells treated with GA-amide at 0, 1, 2, 4, 8, or 12 h (upper) or for 0, 0.1, 0.3, or 1 μM (lower). The statistical results are shown in the right. **g** IP/western blot analysis showing the expression of actin interacting with BMF after treatment with GA-amide in sgNC and sg*WDR1* T2-4 cells. Western blot analysis of cytoplasmic and mitochondrial expression of BMF, BCL2, BAX (**h**), cytochrome C and WDR1 (**i**) and expression in control and *WDR1* knockout cells. **j** A model of the mechanism by which GA-amide inhibited cell invasion and induced cell apoptosis in glioma. The data are presented as the mean ± SEMs (*n* = 3). *P < 0.05, **P < 0.01, ***P < 0.001 compared with the control group by 2-tailed Student’s *t* test in (**b**) and (**f**). For the statistical results in (**h**) and (**i**), one-way ANOVA analysis was appl**i**ed in the comparation of each group

It is worth mentioning that the apoptosis pathway was enriched in the KEGG analysis of the recovered genes in *WDR1* knockout T2-4 cells. Consistent with this finding, GSEA revealed that apoptosis-related genes were positively enriched (Fig. 7a). To confirm the hypothesis that WDR1 might play an essential role in apoptosis induced by GA-amide, control and *WDR1* knockout PDCs were treated with GA-amide or DMSO, and the percentages of apoptotic cells were determined. We found that GA-amide treatment induced apoptosis in control T2-4 cells; however, when *WDR1* was knocked out, the induction of apoptosis by GA-amide was counteracted (Fig. 7b). Notably, we observed that knocking down either MYH9 or CFL1 also resulted in the reversal of the apoptosis induction caused by GA-amide treatment (Supplementary Fig. 11e). Interestingly, the results of the CRISPR/Cas9 screen showed that drug-sensitive genes enriched in the mitochondrial family, especially cytochrome c somatic (CYCS) that encodes cytochrome C, and caspase 9 (CASP9), were also positively enriched in the screen (Fig. 7c–e). Moreover, levels of the apoptosis biomarkers cleaved poly (ADP-ribose) polymerase 1 (PARP) and cleaved caspase3 were increased after GA-amide treatment, which was reversed in *WDR1* knockout cells (Fig. 7f). Thus, we further investigated the mechanism of GA-amide induced apoptosis. It has been reported that once binding with F-actin, a proapoptotic protein Bcl-2-modifying factor (BMF) was inactivated to prevent cell apoptosis.^40^ Based on this research, we applied IP experiments and observed that GA-amide treatment significantly reduced the combination of BMF and actin, which was reversed in the absence of WDR1 (Fig. 7g). After separating and extracting proteins from mitochondria and cytoplasm, we observed a reduction in the cytoplasmic levels of BMF and Bcl2-associated X, apoptosis regulator (BAX), concomitant with an increase in their expression within the mitochondria with GA-amide treatment, which was also reversed in the absence of WDR1 (Fig. 7h). Meanwhile, the anti-apoptotic BCL2 protein was decreased in mitochondria, which was reversed in *WDR1* deficient cells (Fig. 7h). Notably, the activation of BAX leads to its intramembranous oligomerization, forming a proposed pore responsible for the efflux of cytochrome c from mitochondria into the cytosol, which further triggers the cleavage of caspase family members, serving as a crucial mediator in the process of apoptosis.^41,42^ Therefore, we measured the contents of cytochrome C in mitochondria and the cytoplasm after GA-amide treatment in control and *WDR1* knockout PDCs. After treatment with the same concentration of GA-amide, the release of cytochrome C from mitochondria into the cytoplasm was attenuated in *WDR1* knockout cells (Fig. 7i). Collectively, we suggested that GA-amide promoted the destabilization of cytoskeleton *via* directly binding with WDR1, leading to the release of BMF bound to actin, which enabled the translocation of BMF to the mitochondria, triggering the opening of BAX channels and subsequent release of cytochrome C, ultimately inducing apoptosis. (Fig. 7j).

## Discussion

Glioma is a severe malignant disease with limited treatment options and a dismal prognosis, underscoring the critical and pressing need for the exploration of innovative and effective therapeutic approaches. In this study, we presented novel findings demonstrating the anti-glioma effect of GA-amide. Subsequently, we conducted comprehensive investigations to assess the safety, efficacy, and the underlying mechanism of GA-amide. In the development of anticancer chemotherapies, safety is universally recognized as the foremost criterion. In our previous study, we have detected the IC_50_ values of GA-amide in GSCs and neural stem cells (NSCs),^17^ but in this study, we optimized the experimental conditions to obtain a more accurate IC_50_ values. In our in vitro experiments, the IC_50_ values were 0.133 μM–0.399 μM in glioma-related cells, compared with 0.941 μM and 1.03 μM in H1-NSCs and HAs, both of which are normal cells in the CNS. This indicated that GA-amide could specifically target glioma-related cells, while it was a small molecule compound nontoxic to the CNS. The research conducted by Jang and colleagues offered supportive evidence, indicating that GA-amide at concentrations of 10–50 nM exhibited a protective effect on hippocampal neurons against apoptosis triggered by glutamate and induced neurite outgrowth in PC12 cells. Notably, even at a higher concentration of 0.5 μM, GA-amide did not display any toxicity in vitro^18^,which was consistent with our findings. In the BBB permeability assay, GA-amide demonstrated rapid penetration across the BBB. Interestingly, the concentration of GA-amide in the tumor area was found to be two fold higher compared to the non-tumor area, which was quickly decreased in the nontumor area after GA-amide crossed the BBB but did not decrease in the tumor area, making GA-amide more advantageous in terms of tumor targeting and treatment safety.

GA-amide has exhibited notable inhibitory effects on glioma as well, evidenced by the inhibitory of GA-amide on the proliferation of PDCs and GSCs in vitro and the growth of glioma in five glioma mouse models. One of these in vivo models is the transgenic mouse model that closely mimics the characteristics of primary glioma.^43,44^ Furthermore, we applied both the subcutaneous and intracranial orthotopic PDX models to faithfully replicate the heterogeneity found in human gliomas and simulate glioma characteristics observed in patients to a considerable extent.^45,46^ Moreover, we found that GSC tumorigenicity was obviously inhibited in vivo after GA-amide treatment *via* another two mouse models. In addition, our earlier research showed that GA-amide could suppress angiogenesis.^24^ Erin and colleagues found that GA-amide could effectively suppress inflammation and enhance the immune response in breast carcinoma.^23^ Thus, we suspected that GA-amide may have an impact not only on GSCs and GCs but also on the tumor microenvironment, including blood vessels and immune infiltrates. Collectively, these results suggested that GA-amide might be a potential effective and safe chemotherapeutic drug for the treatment of glioma.

Understanding the mechanism of action is a crucial aspect of novel drug research. TrkA was reported as an essential factor for the cellular membrane permeability of GA-amide, as it is indispensable for facilitating the entry of GA-amide into the cytosol. In this research, we found that TrkA was especially highly expressed in glioma tissues, which might explain the preferential activity of GA-amide against tumor cells and indicate that GA-amide may be more effective in patients with high TrkA expression. However, we confirmed that TrkA was not a functional target and was involved only in the entry of GA-amide into the cytoplasm. To investigate the druggable target of GA-amide, genome-wide CRISPR/Cas9 screen was applied to identify drug-sensitive genes and drug resistance genes.^47^ After identifying the drug-sensitive genes that exhibited positive enrichment at the LD80 concentration, three drug target identification technologies were deployed to orthogonally confirm WDR1 as the direct target of GA-amide.

WDR1 exerts direct impact on cell proliferation, migration, and cell communication and maintenance.^48^ Consistent with the results of CRISPR/Cas9 screening, knockout of *WDR1* blunted PDCs responses to GA-amide. We discovered the loss of *WDR1* prevented the inhibition of PDCs invasion by GA-amide. In mechanistic study, we found that the interaction of cytoskeleton-related proteins with WDR1 was enhanced after treatment with GA-amide. As previously reported, WDR1 acts as a major cofactor for Cofilin that plays a crucial role in actin filament turnover, which was achieved by promoting Cofilin-mediated depolymerization of F-actin and regulating the balance between actin depolymerization and assembly.^49^ Actin polymerization and the formation of membrane protrusions are the initial steps for cell migration.^50^ Lee and colleagues found that high expression of WDR1 can promote cell proliferation and migration by regulating dynamic changes in the cytoskeleton in breast cancer.^37^ Moreover, Yuan and colleagues demonstrated that overexpression of WDR1 significantly promoted the migration, invasion and proliferation of non-small cell lung cancer; conversely, WDR1 downregulation suppressed these behaviors.^51^ Here, we revealed that GA-amide induced a reordered distribution of cytoskeleton-related proteins including MYH9 and Cofilin, and then remodeled the F-actin-based cytoskeleton, resulting in inhibition of PDC invasion. More interestingly, we found that GA-amide had a WDR1 dependent proapoptotic effect on PDCs. Notably, it has been reported that WDR1 could promoted the release of BAX-dependent mitochondrial cytochrome C and activation of the caspase-dependent apoptosis cascade in immune cells by interacting with Cofilin.^52^ In this study, we first discovered that GA-amide promoted the destabilization of cytoskeleton *via* directly binding with WDR1 and further assembled with MYH9 and Cofilin as a complex, which released BMF from actin and enabled the translocation of BMF to the mitochondria, triggering the opening of BAX channels and subsequent release of cytochrome C, ultimately inducing apoptosis.

Although our study presented a promising small molecule compound for improving the treatment of glioma, there are several potential limitations. (i) Our study successfully demonstrated the overexpression of TrkA in glioma tissues, but further experiments are required to confirm that TrkA indeed plays a crucial role in facilitating the membrane penetration of GA-amide, not only in PC12 cells but also in GCs. (ii) We have conducted experiments combining GA-amide with clinical drugs for the treatment of glioma. However, the combination of GA-amide and TMZ did not yield a significant improvement in therapeutic efficacy (data not shown). Moving forward, our research will persist in exploring potential combination treatment that can increase the sensitivity to GA-amide.

In summary, our study presented GA-amide as a promising novel option for glioma chemotherapy and revealed its anticancer mechanisms. We have demonstrated that GA-amide directly binds to its druggable target WDR1, leading to the inhibition of GC invasion and induction of WDR1-mediated apoptosis. Specifically, in light of the safety, protective effect and germinal effect associated with GA-amide,^21^ it is possible that combining GA-amide with radiotherapy or chemotherapy could provide enhanced efficacy and mitigating side effects, indicating the clinical potential of GA-amide as a therapeutic agent for glioma. Notably, the negatively selected genes in the CRISPR/Cas9 screen might suggest possible modalities for combined therapy with GA-amide.

## Materials and methods

### Study approval

Glioma samples were obtained after informed consent in the Department of Neurosurgery, Beijing Tiantan Hospital. The normal human brain samples were obtained from the human brain bank of the Institute of Basic Medical Sciences, Chinese Academy of Medical Sciences. The related studies were approved by the institutional review board at Beijing Tiantan Hospital (IRB KY2018-018-02) and the institutional review board of Institute of Basic Medical Sciences, Chinese Academy of Medical Sciences (ZS2023008). Written informed consent was obtained from the patients. All animal studies were approved by the Institutional Animal Care Use & Welfare Committee of the Center for Experimental Animal Research (ACUC-A01-2021-007).

### Cells and cell culture

Four PDC lines (T12-1, T12-2, T2-4, T19-1) obtained from the Beijing Tiantan Hospital^53^ and three GSC lines (GSC2, U87MG-SLC, U251-SLC) were established and verified in our previous work.^17^ Two GCSC lines were kindly provided by Dr. Ran.^54^ These cell lines were cultured in neurobasal medium (Gibco) containing 2% B27 (Gibco), 1% 100 × Antibiotic-Antimycotic (Gibco), 2 mmol/L L-glutamine (catalog SH30034.01, HyClone), 20 ng/mL bFGF (catalog 100-18B-50UG, PeproTech), 20 ng/mL EGF (catalog AF-100-15-100UG, PeproTech), and 10 μg/mL heparin (Sigma‒Aldrich). H1-NSCs obtained from D.Q. Pei’s laboratory (Guangzhou Institutes of Biomedicine and Health, Chinese Academy of Sciences, Guangzhou, China), were cultured in the same medium as the PDCs, with the addition of 1% N-2 supplement (Gibco, Thermo Fisher Scientific). The human astrocyte (HA) cell line was purchased from ScienCell and cultured in commercial astrocyte medium (catalog 1801, ScienCell) supplemented with 1% AGS (ScienCell), 2% FBS (ScienCell) and 1% P/S solution (ScienCell). Regarding the GC cell lines, U87MG cells were purchased from the American Type Culture Collection (ATCC), and U251 cells were purchased from the Cell Center of Peking Union Medical College. The GC cells were cultured in DMEM (Gibco) supplemented with 10% FBS (ScienCell) and 1% 100 × Antibiotic-Antimycotic.

### IC_50_ assay

Cells were seeded in 96-well plates and treated with 0, 0.03, 0.1, 0.3, 1, 3, 10, or 30 μM GA-amide (Santa Cruz) in triplicate. After 48 h of incubation, cell viability was quantified using the MTS assay, and the GA-amide cytotoxicity curve was estimated using GraphPad Prism.

### Tumor sphere formation assay and limiting dilution assay in vitro

For the tumor sphere formation assay, cells were inoculated in 96-well plates at 600 cells/well in a volume of 100 μL. The tumor spheres with a diameter of 50–100 μm and a diameter of over 100 μm were counted separately under a microscope after 14 days. For the limiting dilution assay, cells were dissociated to generate a single-cell suspension, which was diluted along a gradient for seeding in a 96-well plate at concentrations ranging from 400 cells to 25 cells in a volume of 100 μL per well. After 14 days, the percentage of wells that did not contain spheres (diameter ≤50 μm) was recorded, and ELDA method was used to calculate the significance of differences.^55^

### Cell invasion assay

First, cells were starved for 12 h. The top surface of the membrane in the upper transwell chamber was coated with Matrigel (dilution ratio, 1:100, Corning, USA) by incubation for 30 min. Cells were plated in the upper chamber with serum-free neurobasal medium containing the corresponding concentration of GA-amide, and 600 μL of neurobasal medium containing 10% FBS and the same concentration of GA-amide was added to the lower chamber for 24–72 h of culture. Next, the cells in the transwell chamber were fixed with 4% paraformaldehyde for 20 min. After noninvaded cells in the upper chamber were removed by wiping, the remaining cells were stained with 1% crystal violet staining solution for 20 min and washed with double-distilled water. Finally, cell invasion was evaluated by microscopy.

### Western blot analysis

Proteins in the samples were separated on SDS–polyacrylamide gels and transferred to nitrocellulose membranes (Amersham, Sweden). After blocking with 5% skim milk, the membranes were incubated with the indicated primary antibodies listed in Supplementary Table 3 overnight. After washing with 1 × TBST three times, the membranes were incubated with secondary antibodies, and protein bands were detected by using ECL reagent.

### Xenografts

Male nude mice aged 5–6 weeks were selected for establishment of the in vivo glioma mouse models. For the PDX models, the tumor sample will be soaked in HBSS solution on ice and transferred from the hospital to the laboratory. Patient-related information was shown in Supplementary Table 4). For the subcutaneous PDX models, the isometric fresh GBM tumor (GBM1) specimens were implanted s.c. into the flank of a nude mouse. Then, the mice were treated with GA-amide (2 mg/kg i.p.), gambogic acid (2 mg/kg i.p.), TMZ (65 mg/kg i.p., Selleck) or vehicle after the tumors grew to 100 mm^3^. The mouse body weights were measured, and tumor volumes were calculated using the formula L × W^2^/2. After treatment, the xenografts were harvested and photographed.

For the intracranial PDX model, the tumor tissue (GBM2) will be mechanically minced using scissors and then subcutaneously transplanted into the flank of NSG mice (Male, 6–8 weeks). Once the subcutaneous tumors grow, they will be removed and digested with trypsin-EDTA to isolate single cells from the tumor tissue. Then, the digested cells (5 × 10^5^) were injected into the frontal cortex (1 mm rostral to the bregma, 2 mm lateral to the midline, and 3 mm deep) using a microsyringe (Hamilton). After 7 days of tumor development, the mice were grouped dependent on the tumor size measured by MRI. Then 1 mg/kg GA-amide or vehicle was injected i.v. daily for 10 days. After treatment, the tumor size was measured by MRI again.

For the intracranial xenograft mouse models, 1 × 10^5^ GSC2 or U87MG-SLC cells were injected into the frontal cortex (1 mm rostral to the bregma, 2 mm lateral to the midline, and 3 mm deep) using a microsyringe (Hamilton). After 7 days of tumor development, 1 mg/kg GA-amide or vehicle was injected i.v. daily for 13 days. The tumor size was measured by MRI, and survival analysis was performed in a blinded manner.

### BBB permeability assay

Mice bearing U87MG-SLC cell intracranial xenografts were injected with GA-amide (i.v.), and tissues from the brain tumor area and normal brain tissues were collected at different times after injection. For mass spectrometry, a high-resolution accurate mass spectrometer, a Thermo Hypersil Gold C18 HPLC column (5 µm, 50*2.1 mm) coupled to a triple-quadrupole mass spectrometer operated in positive ion mode, the MRM (multiple reaction monitoring) method were used. The diagnostic product ions for GA-amide were detected at m/z 628.3/572.2.

### Genome-wide CRISPR screening

T2-4 cells were infected with lentiviruses carrying the Brunello library (MOI = 0.3). After screening for puromycin (1 µg/mL) resistance, cells were harvested and frozen as DAY0 cells. Other cells were divided into 3 groups and treated with DMSO, 0.25 µM GA-amide (low-dose, LD20) and 0.4 µM GA-amide (high-dose, LD80) for 21 days. Then, the cells were frozen for genomic DNA extraction and Illumina sequencing. The results were analyzed by *MAGeCK* and *MAGeCK-VISPR*.

### Transfection and infection

For cell transfection, we first inserted the full-length cDNA encoding WDR1 and the WDR1 sequence with mutation of the four interaction sites into pcDNA3.1. Then, 293T cells were transiently transfected with these plasmids using Lipofectamine 3000 (Invitrogen). For cell transduction, *WDR1* knockout and WDR1-overexpressing PDCs were generated by lentiviral transduction and selected by 1 µg/mL puromycin. All sg-*WDR1* sequences are listed as follows: sg-*WDR1* #2: 5′-catgggaaatgtgctgacca-3′, sg-*WDR1* #3: 5′-ctgacggtgcataaaaacgg-3′, sg-*WDR1* #4: 5′-gcagcatggacgacaccgtg-3′, sg-*WDR1* #5: 5′-gtgtgcgattctctcctgat-3′. For cell transduction, CFL1 or MYH9 knockdown PDCs were generated by lentiviral transduction and selected by 1 µg/mL puromycin. All shRNA sequences are listed as follows: shCFL1-1: CTATGAGACCAAGGAGAGCAA, shCFL1-2: CCAGATAAGGACTGCCGCTAT, shMYH9-1: GCAAACCTCGAGAAGGCAA, shMYH9-2: GAAGTCAGCTCCCTAAAGA.

### DARTS

The drug affinity responsive target stability (DARTS) method was performed following a previously described procedure.^32^ In brief, cells were collected and lysed with M-PER lysis buffer (catalog 78501, Thermo Fisher Scientific) containing complete protease inhibitor (catalog 04693124001, Roche) and phosphatase inhibitors (catalog 04906845001, Roche). Then, the lysis buffer was supplemented with TNC buffer. The lysates were aliquoted into 1.5 mL tubes and incubated with DMSO or different concentrations of GA-amide for 1 h at room temperature. After incubation, the lysates were digested with 1 μg of pronase for every 300 μg of lysate for exactly 30 min. Then, protein loading buffer was added immediately, and the lysates were heated to stop proteolysis. Western blot analysis was performed for further analysis.

### CETSA

The cellular thermal shift assay (CETSA) has been described previously. Briefly, cells were incubated with DMSO or 3 µM GA-amide for 1 h and washed with PBS three times. Then, the cells were suspended in 1 mL of PBS and divided into equal volumes, followed by heating for 3 min at the indicated temperatures. After heating, three snap freeze–thaw cycles were applied to lyse the cells, and western blotting was performed as previously described.

### SPR

The binding affinities of GA-amide for its target proteins GST-WDR1, GST, and His-WD2-7 were assayed by using the SPR-based Biacore 8 K instrument (GE Healthcare). The CM5 sensor chip was used to immobilize 15,000 RU of the target protein to the sensor surface by the standard amine coupling reaction at 25 °C in PBS running buffer. Gradient concentrations of GA-amide containing 5% DMSO were injected into the channels to evaluate the binding affinity. The dissociation constants (K_D_ values) of the GA-amide–protein complexes were calculated with Biacore 8 K Evaluation Software.

### Recombinant protein expression and purification

The pGEX4T-1 plasmid was used to express the GST-WDR1 and GST proteins, while the pET-28c plasmid was used to express the His-WD2-7 protein. *Arctic-ExpressTM* was used for the production of the GST-WDR1, GST and His-WD2-7 fusion proteins. The cultures were grown at 37 °C, and when the OD_600_ reached 0.5–0.8, the protein was expressed after 0.5 mM IPTG was added. After 4 h, the cells were collected and washed with cold PBS twice.

For GST-WDR1 or GST protein purification, cells were lysed by sonication. The GST protein was present in the supernatant, while the GST-WDR1 protein was present in the precipitate. Precipitates containing GST-WDR1 were solubilized in dissolution buffer (20 mM Tris, 5 mM DTT, 8 M urea, pH 8.0). Proteins were purified on a balanced glutathione-agarose bead-filled column (GE). Next, GST and GST-WDR1 proteins were eluted by GST Elution-Buffer (20 mM Tris-HCl, 50 mM GSH, 0.15 M NaCl, pH 8.0), transferred to a dialysis bag and dialyzed overnight in buffer (20 mM Tris-HCl, 0.15 M NaCl, pH 8.0).

For His-WD2-7 protein purification, cells were first lysed by sonication. WD2-7 was present the precipitate and solubilized as described above. Then, the protein was loaded onto a Balanced Ni-IDA-Sepharose Cl-6B column (Novagen) and washed with Ni-IDA Washing-Buffer (20 mM Tris-HCl, 20 mM imidazole, 0.15 M NaCl, pH 8.0). Then, the protein was eluted with Ni-IDA Elution-Buffer (20 mM Tris-HCl, 250 mM imidazole, 0.15 M NaCl, pH 8.0), transferred to a dialysis bag and dialyzed overnight in PBS buffer.

### Apoptosis assay

An apoptosis detection kit (KeyGEN BioTECH) was used for the PDC apoptosis assay. After treatment with different concentrations of GA-amide, cells were collected and resuspended in 100 µL of binding buffer with 1 µl FITC Annexin V and 1 µl PI for 15 min at room temperature in the dark. The cell suspensions were analyzed by flow cytometry within 1 h.

### F-actin isolation and detection

F-actin isolation was performed according to the instructions of the G-actin/F-actin assay (Cytoskeleton, Inc.). Briefly, cells were lysed in LAS2 buffer (Cytoskeleton, Inc.). The lysates were centrifuged (5 min at 1000 rpm) to pellet undisrupted cells, and then 100 µl of the supernatant was ultracentrifuged at 37 °C (100,000 × g) for 1 h to separate F-actin from soluble G-actin. The F-actin pellet was solubilized by incubation in 100 μL of F-actin depolymerization buffer for 1 h. The soluble F-actin and G-actin fractions were subsequently analyzed by immunoblotting with a mouse monoclonal anti-actin antibody (Cytoskeleton, Inc.).

### Statistics and data interpretation

Mean values and ± SEMs are shown unless otherwise indicated. Student’s *t* test or one-way ANOVA was used as appropriate to analyze data from in all in vitro and in vivo experiments as indicated in the figure legends. IC_50_ calculations were performed and line plots and bar charts were generated using GraphPad Prism. Differences in Kaplan‒Meier survival curves were analyzed by the Mantel‒Cox log–rank test. The stem cell self-renewal assay results were analyzed using ELDA. The MRI images of the brain and images of cell invasion were quantitatively analyzed by ImageJ. The CRISPR/Cas9 screening data were analyzed by *MAGeCK* and *MAGeCK-VISPR*. GO and KEGG pathway analyses were carried out using the “clusterProfiler” package in R (R version 4.1.1).

### Supplementary information

Supplementary materials

Dataset 1

## Data Availability

The authors confirm that all the data supporting the findings of this study are included in the article and its Supplemental Information files. The raw sequence data reported in this paper have been deposited in the Genome Sequence Archive^56^ in National Genomics Data Center,^57^ China National Center for Bioinformation / Beijing Institute of Genomics, Chinese Academy of Sciences (GSA-Human: HRA005504) that are publicly accessible at https://ngdc.cncb.ac.cn/gsa-human. The mass spectrometry proteomics data have been deposited to the ProteomeXchange Consortium *via* the PRIDE^58^ partner repository with the dataset identifier PXD045343. Any additional materials related to this article can be obtained upon reasonable request from the corresponding authors.
