# Supplementary material for: The tumor-enriched small molecule gambogic amide suppresses glioma by targeting WDR1-dependent cytoskeleton remodeling
Source: Signal Transduct Target Ther. 2023 Nov 8;8:424. doi: 10.1038/s41392-023-01666-3 (PMC10630452; doi:10.1038/s41392-023-01666-3)
Supplement: Supplementary file 1 — Supplementary materials [file 41392_2023_1666_MOESM1_ESM.docx]

Supplementary Materials for

The tumor-enriched small molecule gambogic amide suppresses glioma by targeting WDR1-dependent cytoskeleton remodeling

Jiaorong Qu ^1, 2 †^, Bojun Qiu ^1, 2 †^, Yuxin Zhang ^1, 2^, Yan Hu ^1, 2^, Zhixing Wang ^1, 2,^ Zhiang Guan ^1, 2^, Yiming Qin ^1, 2^, Tongtong Sui ^1, 2^, Fan Wu ^3, 4^, Boyang Li ^1, 2^, Wei Han ^1, 2 *^, Xiaozhong Peng ^1, 5, 6 *^

Correspondence to: pengxiaozhong@pumc.edu.cn, hanwei2012@ibms.pumc.edu.cn

**This PDF file includes:**

Materials and Methods

Supplementary Text

Figures. S1 to S11

Tables S1 to S4

Captions for Data S1

**Other Supplementary Materials for this manuscript include the following:**

Data S1 [Positive enriched genes in CRISPR/Cas9 screening]


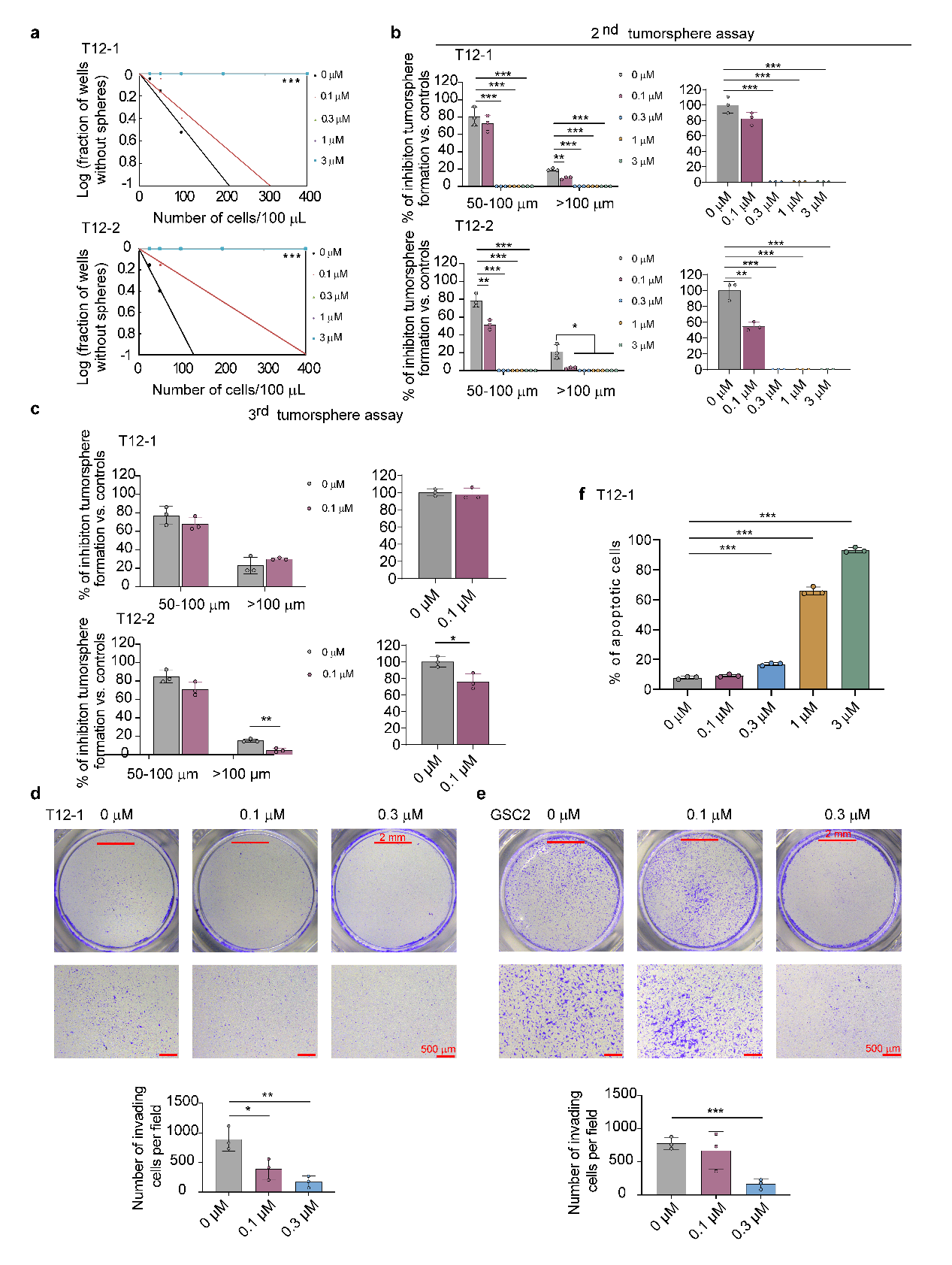


**Supplementary Fig. 1** **GA-amide inhibited malignant characteristics of PDCs *in vitro*. a** Limiting dilution assay of PDCs (T12-1, T12-2) after pre-treatment with GA-amide for 4 hours. For PDCs, the three lines that represent the treatment of 0.3 μM, 1 μM and 3 μM were overlapped. Data were analyzed by ELDA, n = 10 for each group. **b** Secondary tumorsphere assay of PDCs (T12-1, T12-2) after pretreatment with GA-amide for 4 hours. **c** Tertiary tumorsphere assay of PDCs (T12-1, T12-2) collected from second tumorspheres that pretreated with GA-amide for 4 hours. **d, e** The images and statistical results of the transwell invasion assays in T12-1 (**d**) and GSC2 (**e**) cell with the treatment of GA-amide for 48 hours. Scale bars: 2 mm (upper pannel); 500 μm, (lower panel). **f** Flow cytometry–based quantification of T12-1 cells apoptosis after 4 hours of exposure at different concentrations of GA-amide treatment by annexin V/PI staining. For (**b**-**f**), data were presented as the mean ± SEM (n = 3) and analyzed by ANOVA, **P* < 0.05, ***P* < 0.01, ****P* < 0.001.

**
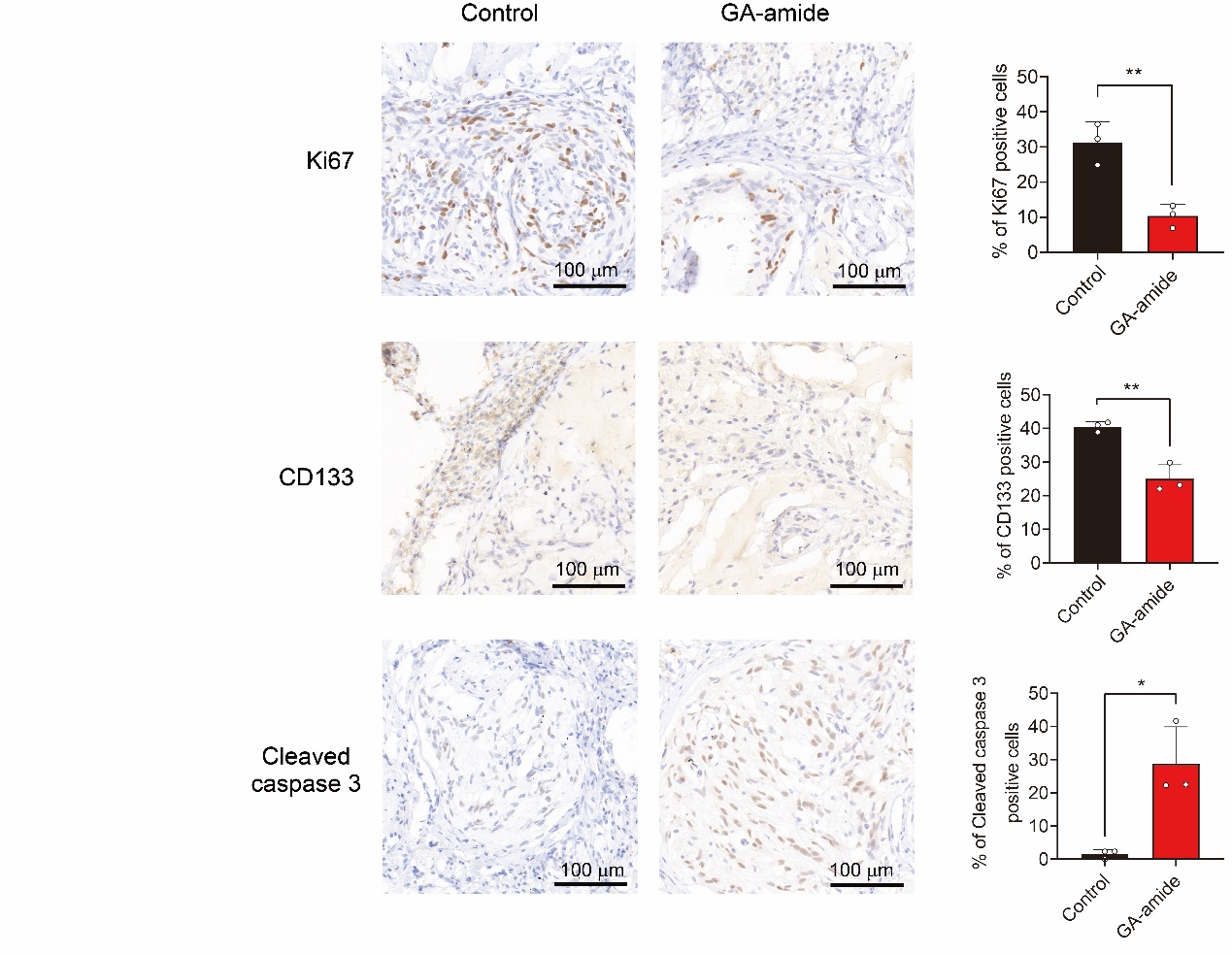
**

**Supplementary Fig. 2** **Antitumor effects of GA-amide in xenograft tumor experiments.** Ki67, CD133 and Cleaved caspase 3-stained sections (scale bars: 100 μm) of PDX tumors after treatments of control (DMSO) or GA-amide for 10 days. Data were calculated as the mean ± SEM (n = 3) and analyzed by 2-tailed Student’s t test, **P* < 0.05, ***P* < 0.01.


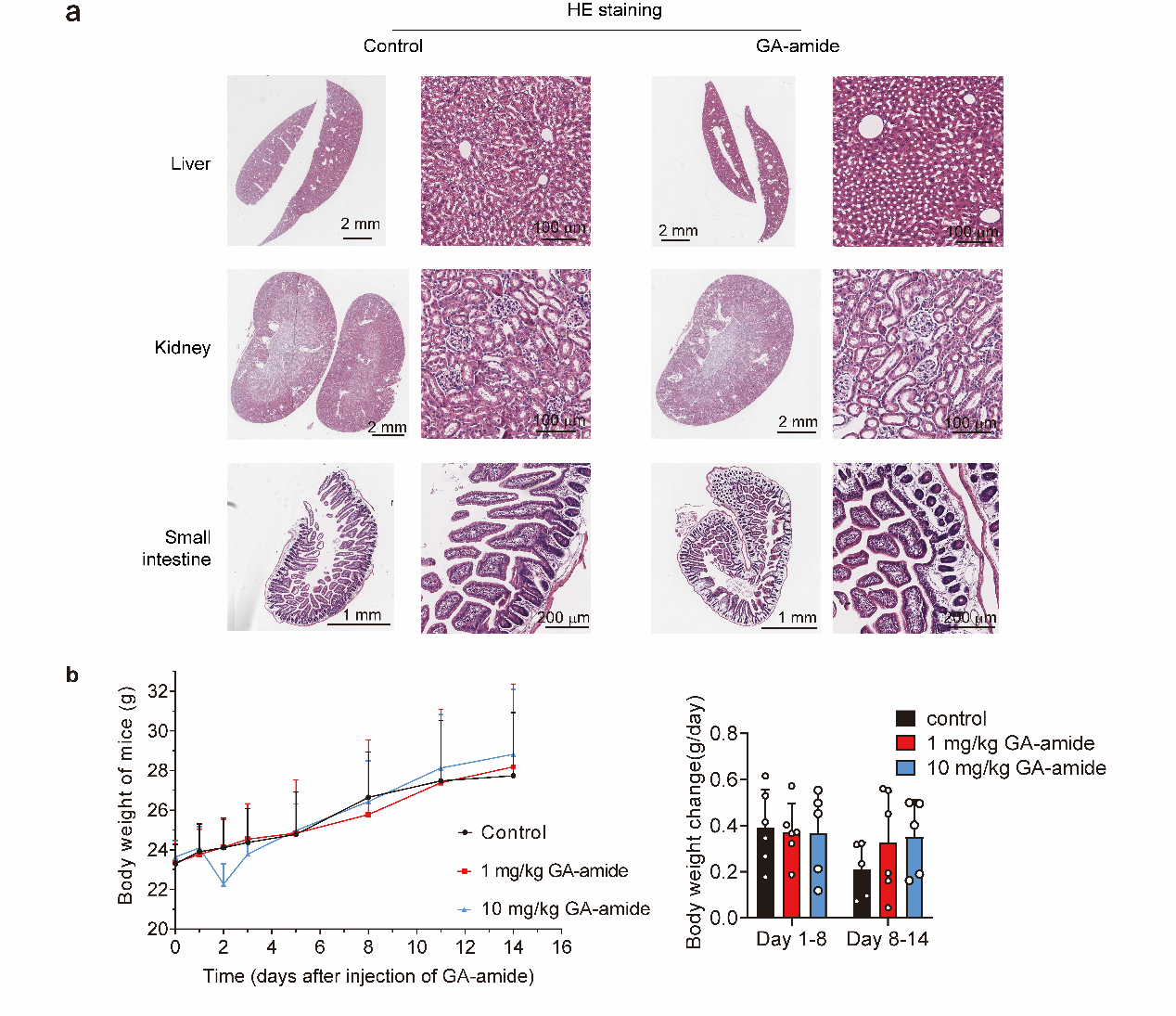


**Supplementary Fig. 3** **Safety assessments of GA-amide *in vivo* treatment.**

**a** Hematoxylin and eosin staining of sections from control and GA-amide-treated livers, kidney and small intestines, scale bars were shown in the panel. **b** Persistent records of the body weights and the statistical results of the body weight changes after one-time i.v. treatments of control (DMSO), 1 mg/kg GA-amide and 10 mg/kg GA-amide (n = 6, for control and 1mg/kg GA-amide; n = 5, for 10mg/kg GA-amide) were shown.


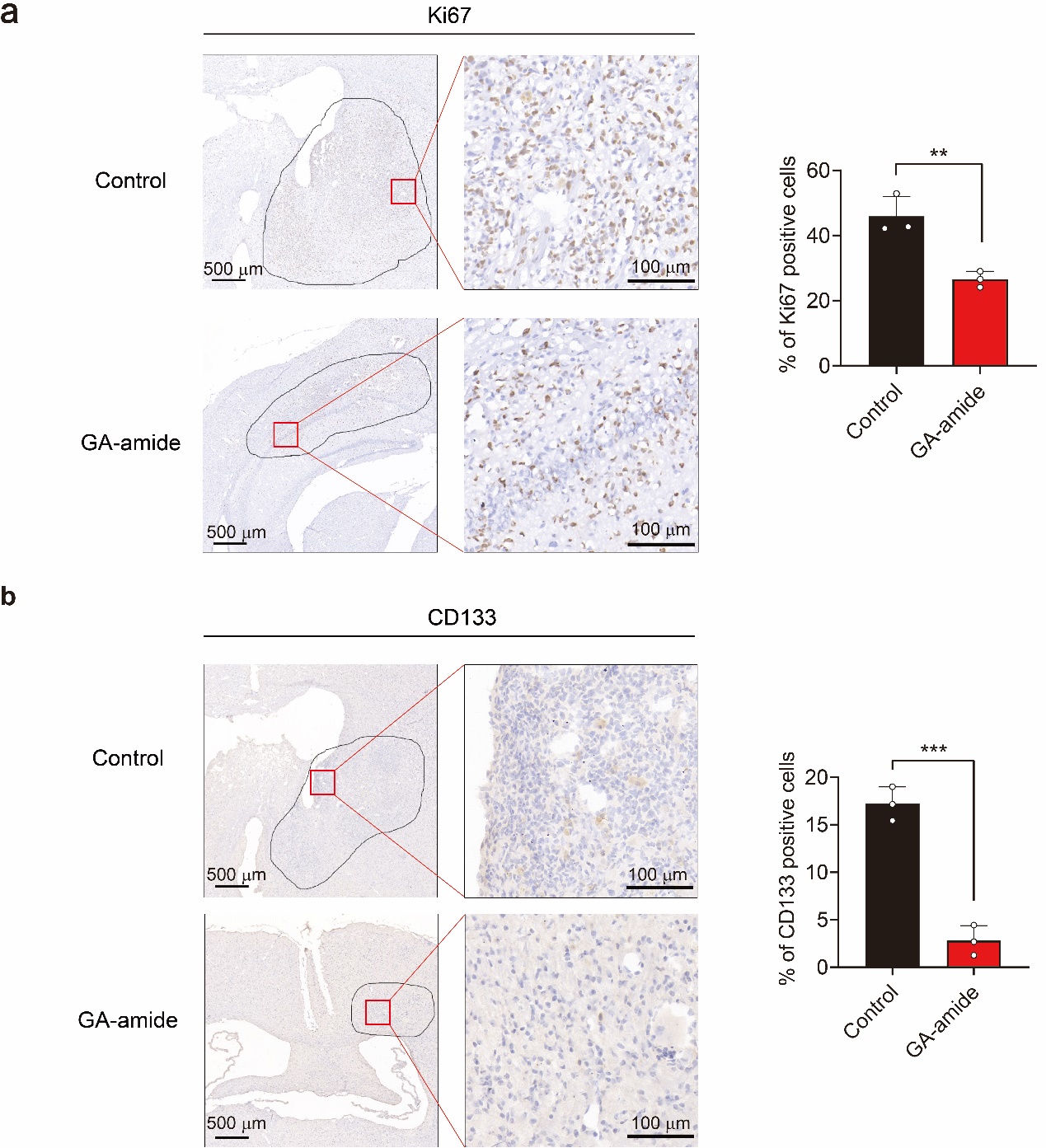


**Supplementary Fig. 4** **Antitumor effects of GA-amide in transgenetic xenografts experiments.** **a, b** Immunohistochemical analysis of sections from tumors induced by injection with pTomo-Ras-sip53 lentivirus after GA-amide treatment, which was stained with antibody against Ki67 (**a**) and CD133 (**b**), scale bars: 500 μm and 100 μm. Data were calculated as the mean ± SEM (n = 3) and analyzed by 2-tailed Student’s t test, ***P* < 0.01, ****P* < 0.001.


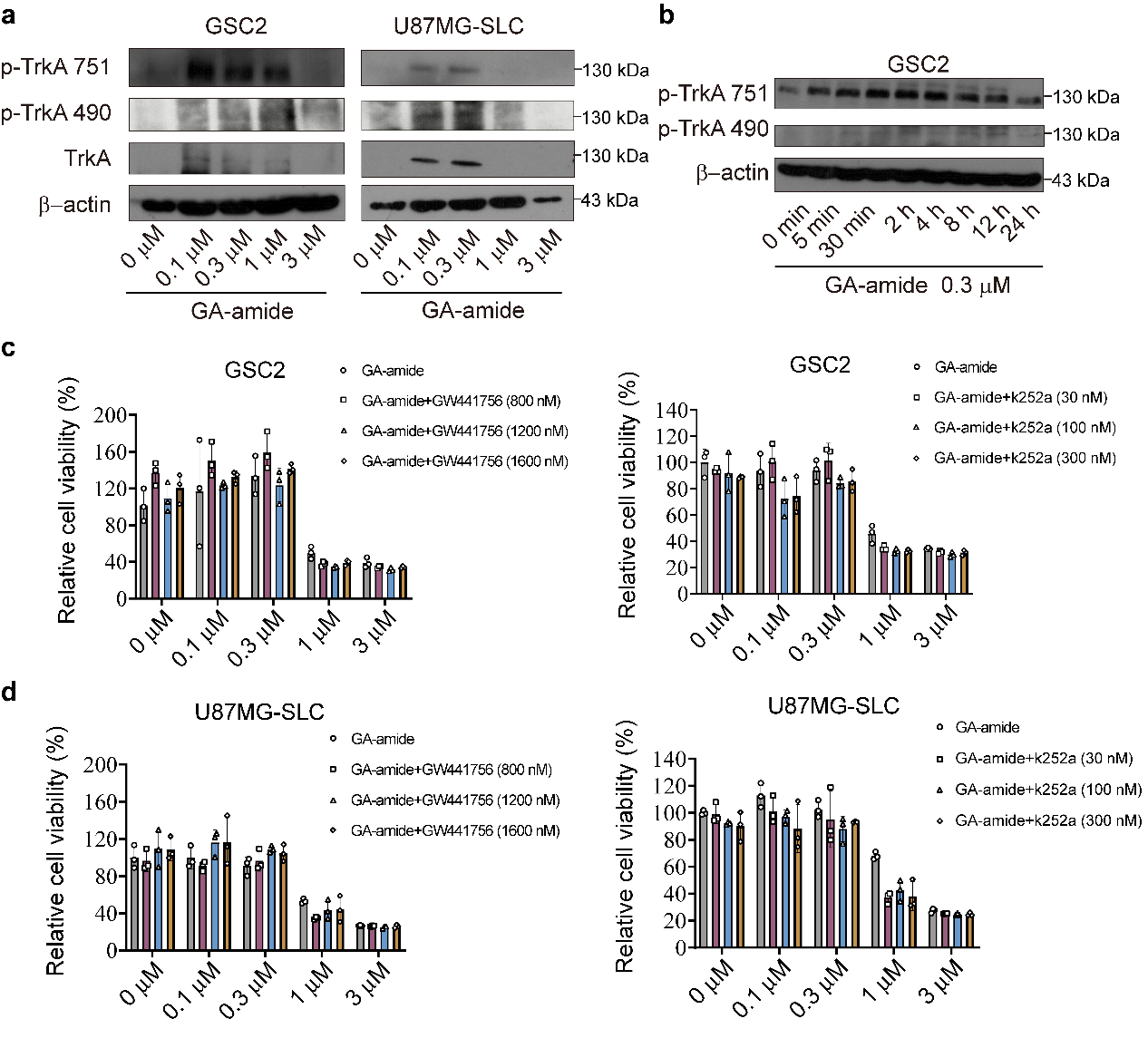


**Supplementary Fig. 5 TrkA was not the functional target for GA-amide to inhibit glioma. a** Western blot analyses of the TrkA and p-TrkA expression in GSC2 and U87MG-SLC cells with GA-amide treatment for 4 hours. **b** Western blot analyses of the p-TrkA expression in GSC2 with GA-amide treatment for different times. **c, d** The combination effects of GA-amide with GW441756, a TrkA inhibitor, or with K252a, an inhibitor of Trk family in GSC2 (**c**) and U87MG-SLC (**d**) (48 hours, n = 3).

**
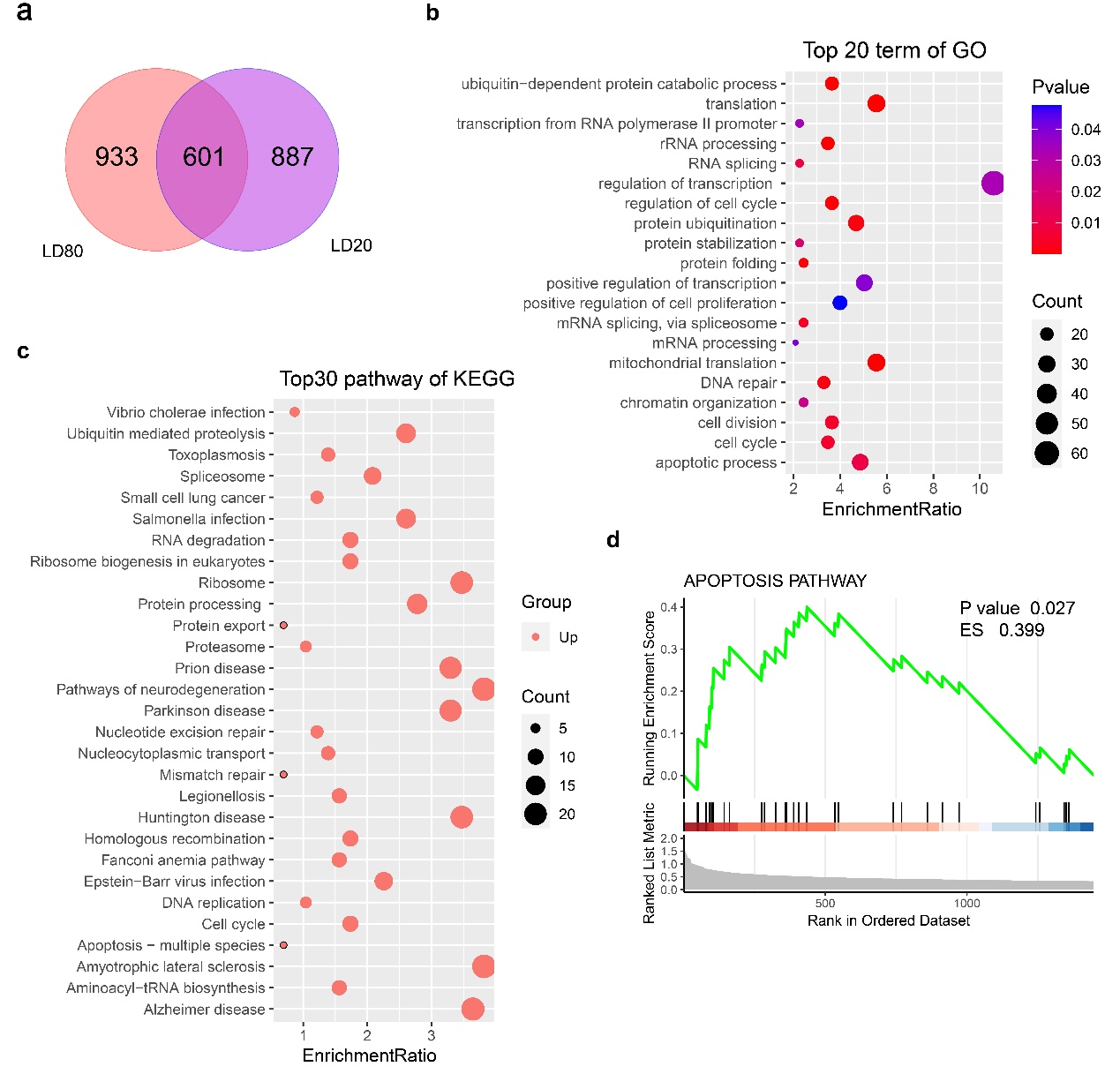
**

**Supplementary Fig. 6 The enrichment analysis of the CRISPR/Cas9 screening. a** Venn diagram showed the overlap of CRISPR/Cas9 positively selected genes between LD20 treatment group and LD80 treatment group. **b**, **c** GO (**b**) and KEGG (**c**) analysis of the 601 positively selected genes. **d** GSEA analysis showed that apoptosis pathway was enriched.


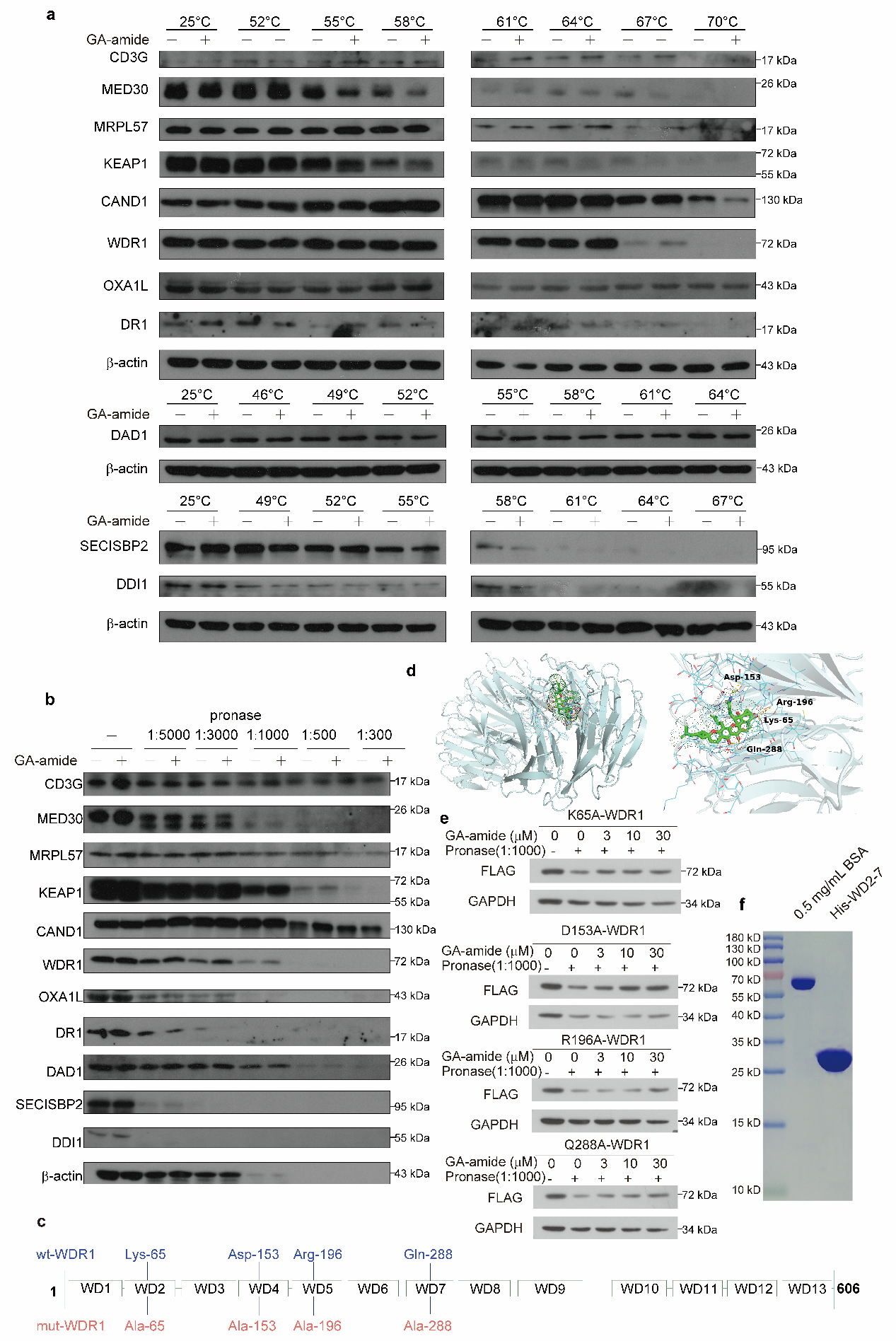


**Supplementary Fig. 7** **WDR1 was identified as the druggable target among the candidate genes screened by CRISPR. a** Western blot analysis of the CETSA samples of T2-4 cells. **b** Western blot analysis of the DARTs samples of T2-4 cells. **c, d** Molecular docking software was used to predict the interaction between WDR1 and GA-amide. The secondary structure of WDR1, in which the four binding sites and the mutant sites were shown (**c)**. The binding affinity between WDR1 and GA-amide was -10.7 Kcal/mol (**d**). **e** Western blot analysis showed the protection of GA-amide for the single residue mutant WDR1 with pronase treatment in the DARTS assay in 293T cells. **f** Purity analysis of purified fusion His-WD2-7 protein by Coomassie staining.


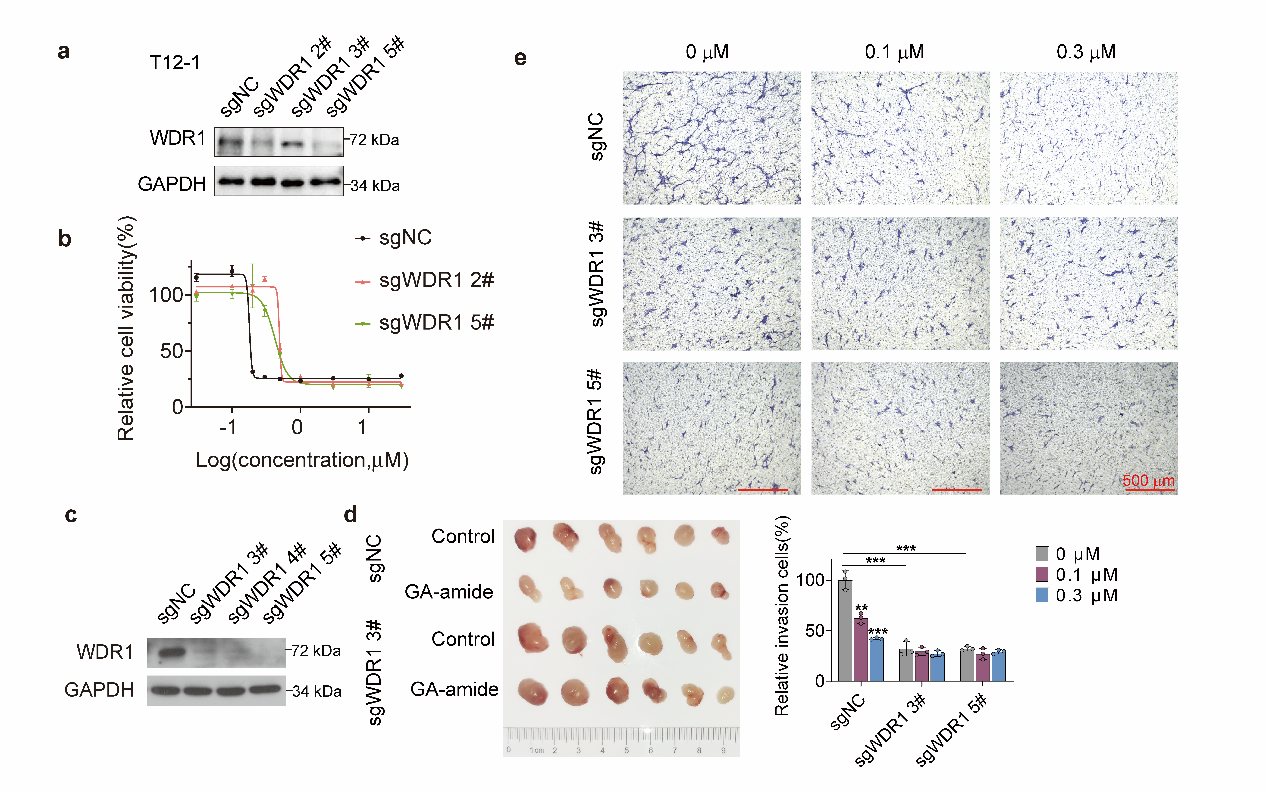


**Supplementary Fig. 8 Knockout of WDR1 was able to recover GA-amide inhibition. a** The knockout effects of sgRNAs targeting WDR1 in T12-1 cells. **b** The cell viability assay for the WDR1 deficient T12-1 cells with different dose of GA-amide treatment. **c** The knockout effects of sgRNAs targeting WDR1 in U87MG-SLC cells. **d** The tumor images of mice injected with sgNC and sgWDR1 3# U87MG-SLC cells were captured after being treated with DMSO or GA-amide (2 mg/kg) *via* intraperitoneal (i.p.) injection once daily for a duration of 10 days. **e** Transwell assay for WDR1 deficient T12-1 cells treated with 0 μM, 0.1 μM and 0.3 μM GA-amide. The images were representative of 3 wells, scale bar: 500 μm. Data were calculated as the mean ± SEM (n = 3) and analyzed by a one-way ANOVA analysis, **P* <0.05, ***P* <0.01 and ****P* <0.001.


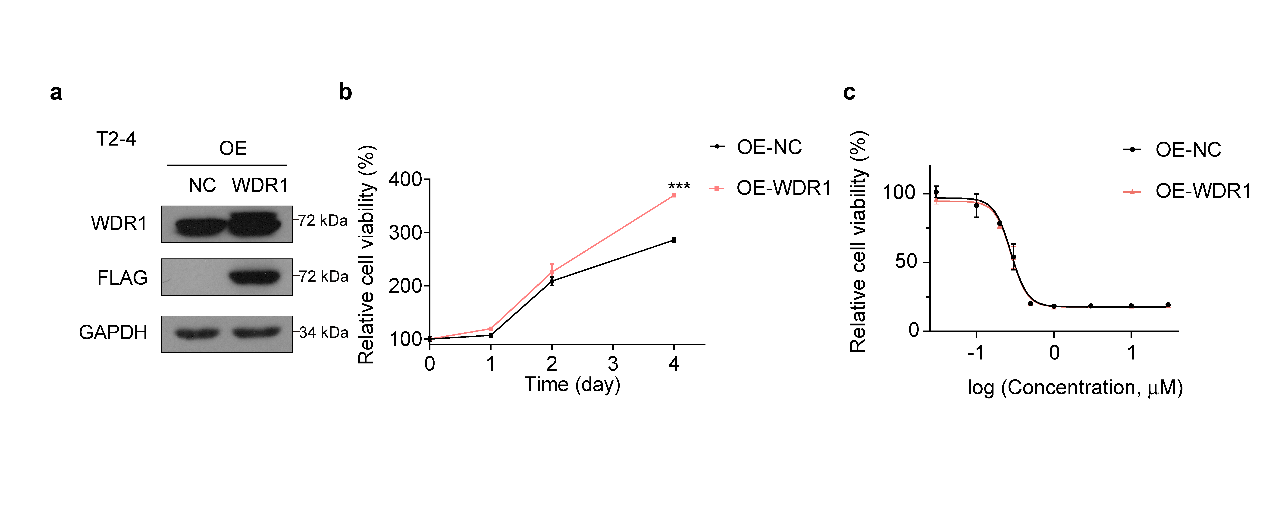
**Supplementary Fig. 9** **Overexpression of WDR1 could not sensitize PDCs to GA-amide. a** Western blot analyses of FLAG-WDR1 overexpression in T2-4 cells. **b** The relative cell viability of OE-NC and OE-WDR1 T2-4 cells were detected at 0, 24, 48, and 96 hours by MTS, samples were assayed in triplicate. **c** OE-NC and OE-WDR1 T2-4 cells were treated with the indicated concentrations of GA-amide for 48 hours, and viability was measured by MTS, samples were assayed in triplicate.


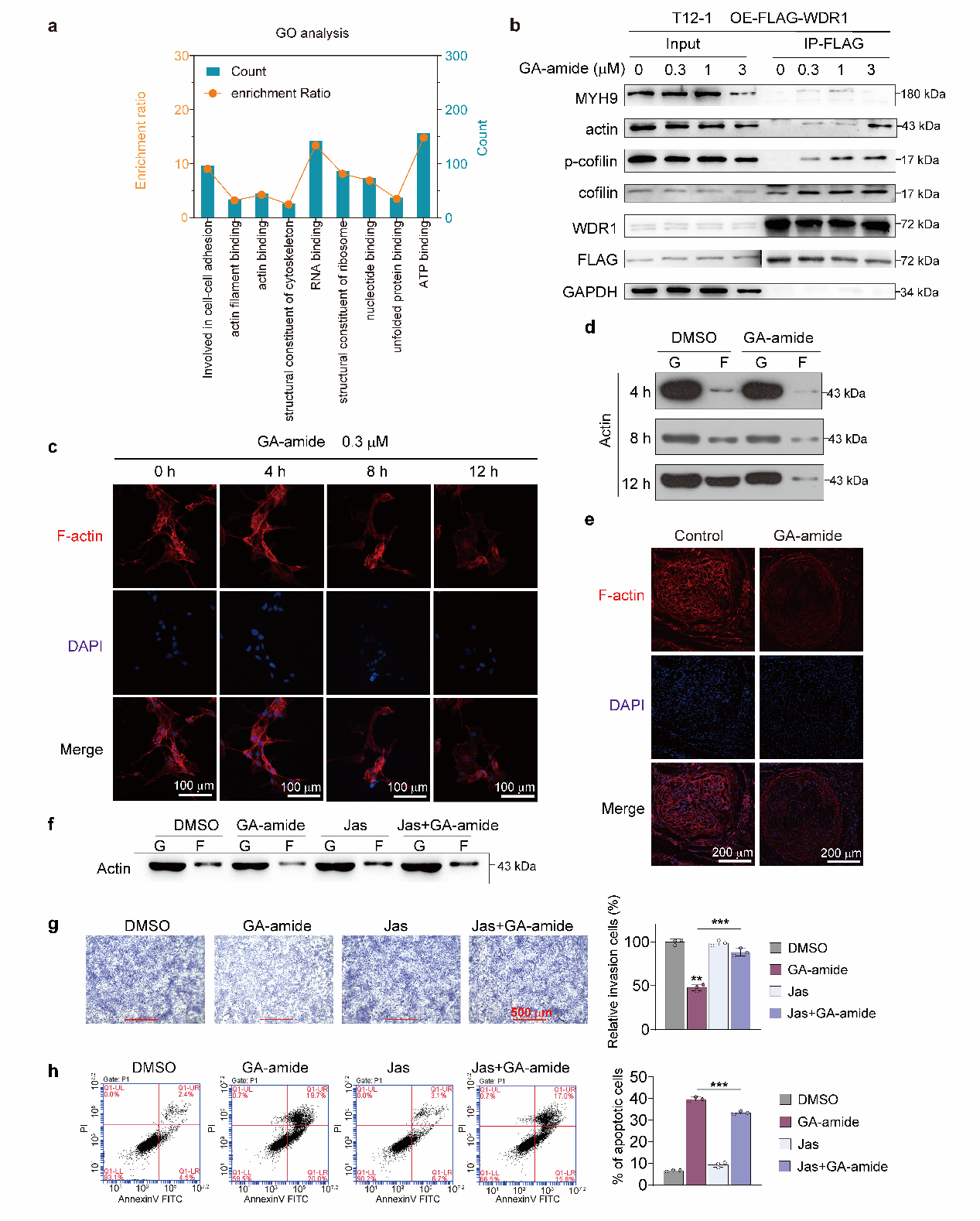


**Supplementary Fig. 10 GA-amide strengthened the combination of WDR1 with cytoskeleton related proteins and disrupted cytoskeleton remodeling. a** GO enrichment analysis of WDR1 combined proteins after 3 μM GA-amide treated for 1 hour in IP-MS. **b** Western blot analyses showed the expression of cytoskeleton related proteins combined with WDR1 after different GA-amide treated in T12-1 cells. **c** Phalloidin-TRITC visualization of F-actin (red), DAPI staining of DNA (blue) and merged images of T2-4 cells with 0-, 4-, 8- and 12-hours treatment of 0.3 μM GA-amide treatment. scale bar: 100 μm. **d** Western blot analyses of the isolated F-actin and G-actin from the T2-4 cells treated with DMSO or GA-amide for 4, 8, 12 hours, blotted with anti-actin. **e** Phalloidin-TRITC visualization of F-actin (red) and DAPI staining of DNA (blue) and merged images of sections from PDX tumors after treatments of control (DMSO) or GA-amide for 10 days. scale bar: 200 μm. **f** F-actin and G-actin extracted from T2-4 cells treated with DMSO, GA-amide (0.3 μM), jas (50 nM), and jas (50 nM jas was pretreated for 12 hours) in combination with GA-amide (0.3 μM) for 8 hours were analyzed through western blotting. **g** Transwell assay of T2-4 cells treated with DMSO, GA-amide (0.3 μM), jas (50 nM), and jas (50 nM) in combination with GA-amide (0.3 μM) for 48 hours. Representative images of three wells are shown, and the scale bar represents 500 μm. **h** The apoptosis assay was conducted on T2-4 cells treated with DMSO, GA-amide (0.3 μM), jas (50 nM), and jas (50 nM jas was pretreated for 12 hours) in combination with GA-amide (0.3 μM) for 4 hours. The data were presented as the mean ± SEM (n = 3) and analyzed using one-way ANOVA analysis. Statistical significance is denoted as **P* <0.05, ***P* <0.01, and ****P* <0.001.


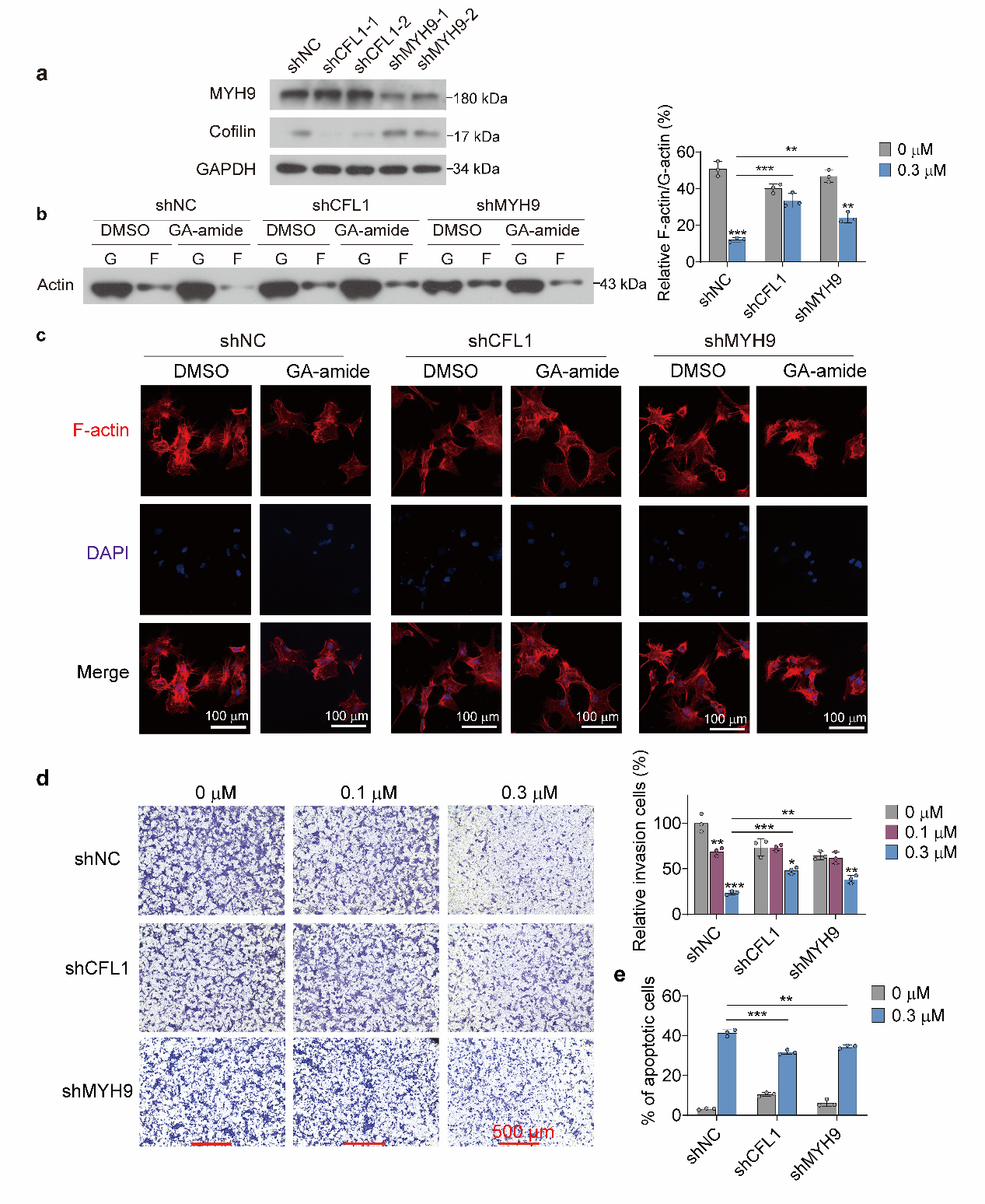


**Supplementary Fig. 11 Knockdown of CFL1 or MYH9 reversed the inhibitory effect of GA-amide. a** The knockdown effects of shRNAs targeting CFL1 and MYH9 in T2-4 cells. **b** F-actin and G-actin extracted from shNC, shCFL1-1 and shMYH9-1 T2-4 cells treated with DMSO or GA-amide for 8 hours were subjected to immunoblotting with an anti-actin antibody. The statistical results showed in the right. **c** TRITC-phalloidin staining of F-actin (red), DAPI staining of DNA (blue), and merged images in shNC, shCFL1-1 and shMYH9-1 T2-4 cells after 0.3 μM GA-amide treatment for 8 hours, scale bar: 100 μm. **d** Transwell assays showed that shCFL1-1 or shMYH9-1 prevented the inhibition of invasion by GA-amide in T2-4 cells. The data are representative of 3 wells, scale bar: 500 μm. **e** Knockdown of CFL1 or MYH9 suppressed the induction of apoptosis after 0.3 μM GA-amide treatment for 4 h. Data were calculated as the mean ± SEM (n = 3) and analyzed by 2-tailed Student’s t test, **P* <0.05, ***P* <0.01 and ****P* <0.001.

**Supplementary Tables**

| Supplementary Table 1. **Blood routine examination** | | | | | | |  |
| --- | --- | --- | --- | --- | --- | --- | --- |
| Analytes | | Control (n = 6) | | 1mg/kg GA-amide (n = 6) | | 10mg/kg GA-amide (n = 5) |  |
| WBC, ×103cells/μL | | 4.36±1.02 | | 4.78±1.34 | | 4.38±0.95 |  |
| NEUT, ×103cells/μL | | 0.91±0.39 | | 1.05±0.51 | | 0.81±0.29 |  |
| LYMPH, ×103cells/μL | | 3.15±0.68 | | 3.48±0.95 | | 3.18±0.75 |  |
| MONO, ×103cells/μL | | 0.08±0.06 | | 0.06±0.03 | | 0.05±0.03 |  |
| EOS, ×103cells/μL | | 0.21±0.05 | | 0.18±0.08 | | 0.33±0.24 |  |
| BASO, ×103cells/μL | | 0.00±0.00 | | 0.00±0.00 | | 0.00±0.00 |  |
| LUC, ×103cells/μL | | 0.02±0.01 | | 0.02±0.01 | | 0.01±0.00 |  |
| NEUT, % | | 20.3±6.3 | | 21.8±7.5 | | 18.1±4.6 |  |
| LYMPH, % | | 72.8±6.1 | | 73.0±6.2 | | 72.4±3.7 |  |
| MONO, % | | 1.8±0.8 | | 1.1±0.5 | | 1.0±0.4 |  |
| EOS, % | | 4.8±1.3 | | 3.8±1.5 | | 8.1±7.2 |  |
| BASO, % | | 0.0±0.0 | | 0.0±0.0 | | 0.0±0.0 |  |
| LUC, % | | 0.4±0.1 | | 0.4±0.1 | | 0.3±0.1 |  |
| RBC, ×106cells/μL | | 8.68±0.44 | | 9.09±1.40 | | 10.13±1.05 |  |
| HGB, g/dL | | 13.3±0.6 | | 13.0±0.7 | | 13.6±0.5 |  |
| HCT, % | | 41.1±2.5 | | 42.8±7.5 | | 48.5±5.1 |  |
| MCV, fL | | 47.4±1.6 | | 47.0±1.8 | | 47.8±1.1 |  |
| MCH, pg | | 15.3±0.7 | | 14.5±1.7 | | 13.6±1.8 |  |
| MCHC, g/dL | | 32.3±1.3 | | 31.0±3.9 | | 28.4±3.8 |  |
| RDW, % | | 16.2±1.0 | | 16.5±1.2 | | 17.0±0.6 |  |
| HDW, g/dL | | 2.62±0.09 | | 2.66±0.20 | | 2.71±0.10 |  |
| CHCM, g/dL | | 35.6±0.5 | | 35.7±0.8 | | 35.4±1.3 |  |
| CH, pg | | 16.9±0.6 | | 16.8±0.5 | | 17.0±0.5 |  |
| CHDW, % | | 2.90±0.14 | | 3.00±0.39 | | 3.15±0.11 |  |
| PLT, ×103cells/μL | | 1548±312 | | 1603±310 | | 1936±670 |  |
| PCT, % | | 1.28±0.29 | | 1.33±0.30 | | 1.63±0.60 |  |
| PDW, % | | 48.4±1.5 | | 48.8±2.5 | | 49.9±2.3 |  |
| MPV, fL | | 8.2±0.3 | | 8.3±0.3 | | 8.4±0.2 |  |
| #Retic, ×109cells/L | | 337.2±107.0 | | 335.1±72.9 | | 836.7±674.4 |  |
| Notes: WBC: White Blood Cell, NEUT: Neutrophil, LYMPH: Lymphocyte, MONO: Monocyte, EOS: Eosinophil, BASO: Basophil, LUC: Large Unstained Cell, RBC: Red Blood Cell, HGB: Hemoglobin, HCT: Hematocrit, MCV: Mean Corpuscular Volume, MCH: Mean Corpuscular Hemoglobin, MCHC: Mean corpuscular hemoglobin concentration, RDW: red blood cell volume distribution width, HDW: Hemoglobin Distribution Width, CHCM: Cellular Hemoglobin Concentration Mean, CH: corpuscular hemoglobin, CHDW: Cell hemoglobin distribution width, PLT: Platelet, PCT: Plateletocrit, PDW: Platelet distribution width, MPV: Mean platelet volume, Retic: Reticulocyte. | | | | | | |  |
|  |  |  |  |  |  |  |  |
|  |  |  |  |  |  |  |  |
|  |  |  |  |  |  |  |  |
|  |  |  |  |  |  |  |  |
| Supplementary Table 2. **Mice serum biochemical indexes** | | | | | | | |
| Analytes | Contrl (n = 6) | | 1mg/kg GA-amide (n = 6) | | 10mg/kg GA-amide (n = 5) | | |
| ALT (U/L) | 34±6 | | 26±2* | | 37±12 | | |
| AST (U/L) | 106±21 | | 99±20 | | 113±22 | | |
| TP (g/L) | 58.4±3.0 | | 54.5±6.7 | | 55.5±1.8 | | |
| ALB (g/L) | 21.2±1.4 | | 19.5±2.4 | | 20.0±0.9 | | |
| GGT (U/L) | -1±1 | | -1±1 | | -1±1 | | |
| ALP (U/L) | 251±48 | | 214±36 | | 217±33 | | |
| UREA (mmol/L) | 8.16±1.66 | | 8.11±1.58 | | 8.62±1.32 | | |
| CRE (μmol/L) | 12.4±1.2 | | 11.1±1.1 | | 10.9±0.6 | | |
| TBA (μmol/L) | 17.4±7.6 | | 8.6±6.4 | | 7.9±4.5 | | |
| TCHO (mmol/L) | 4.68±0.59 | | 4.34±1.10 | | 4.75±0.93 | | |
| TG (mmol/L) | 1.87±0.69 | | 1.30±0.29 | | 1.62±0.48 | | |
| GLU (mmol/L) | 6.82±0.72 | | 8.21±3.50 | | 5.61±0.60 | | |
| CK (U/L) | 832±420 | | 910±503 | | 1157±539 | | |
| CHE (U/L) | 8073±828 | | 7909±1092 | | 7828±721 | | |
| CA (mmol/L) | 2.63±0.07 | | 2.53±0.12 | | 2.54±0.07 | | |
| TBIL (μmol/L) | 4.1±0.5 | | 3.3±0.5 | | 3.8±1.0 | | |
| K (mmol/L) | 5.83±0.57 | | 5.99±0.84 | | 5.97±0.33 | | |
| NA (mmol/L) | 147.5±1.3 | | 147.4±1.5 | | 147.7±2.0 | | |
| Cl (mmol/L) | 108.1±3.7 | | 112.0±2.2* | | 111.5±1.7 | | |
| **P* < 0.05, ***P* < 0.01, ****P* < 0.001 compared with control group by 2-tailed Student’s t test. | | | | | | | |
|  |  |  |  |  |  |  |  |
| ALT: alanine aminotransferase, AST: aspartate aminotransferase, TP: total protein, ALB: albumin, GGT: Gammagliutamyltranspherase, ALP: alkaline phosphatase, UREA: Urea, CRE: creatinine, TBA: total bile acid, TCHO: total cholesterol, TG: triglyceride, GLU: glucose, CK: creatine kinase, CHE: cholinesterase, CA: calcium, TBIL: total bilirubin. | | | | | | | |
|  |  |  |  |  |  |  |  |
|  |  |  |  |  |  |  |  |
|  |  |  |  |  |  |  |  |
|  |  |  |  |  |  |  |  |

| Supplementary Table 3. **Antibody information** | | | |
| --- | --- | --- | --- |
| Antibodies | Source | Identifier | Dilution |
| CAND1 (D1F2) Rabbit mAb | CST | #8759 | 1:1000 |
| CD3 Gamma Monoclonal Antibody | Proteintech | 60347-1-Ig | 1:300 |
| Anti-DR1 | Abcam | ab88597 | 1:1000 |
| Anti-Keap1 | Abcam | ab139729 | 1:1000 |
| WDR1 Polyclonal Antibody | Proteintech | 13676-1-AP | 1:1000 |
| MED30 Monoclonal Antibody | Proteintech | 67038-1-Ig | 1:1000 |
| DAD1 Polyclonal Antibody | Proteintech | 10531-1-AP | 1:1000 |
| OXA1L Monoclonal Antibody | Proteintech | 66128-1-Ig | 1:1000 |
| SECISBP2 Polyclonal Antibody | Proteintech | 12798-1-AP | 1:500 |
| DDI1 Polyclonal Antibody | Proteintech | 13968-1-AP | 1:500 |
| MRPL57 Polyclonal Antibody | Invitrogen | PA5-66250 | 1:1000 |
| Ubiquilin 1 Polyclonal Antibody | Proteintech | 23516-1-AP | 1:300 |
| Phospho-Cofilin (Ser3) (77G2) Rabbit mAb | CST | 3313T | 1:500 |
| Cofilin Polyclonal Antibody | Proteintech | 10960-1-AP | 1:1000 |
| GAPDH Monoclonal Antibody | Proteintech | 60004-1-Ig | 1:10000 |
| Rabbit polyclonal anti-PARP | CST | 9542L | 1:1000 |
| Rabbit monoclonal anti-Caspase 3 | CST | 9665S | 1:500 |
| Rabbit monoclonal anti-Cleaved caspase 3 | CST | 9661L | 1:200 |
| Beta Actin Monoclonal Antibody | Proteintech | 66009-1-Ig | 1:10000 |
| Anti-TrkA | Santa Cruz | sc-118 | 1:100 |
| p-TrkA490 | CST | 9141S | 1:1000 |
| p-TrkA751 | Invitrogen | 1083024B | 1:1000 |
| Cytochrome c Monoclonal antibody | Proteintech | 66264-1-Ig | 1:500 |
| COXIV Polyclonal antibody | Proteintech | 11242-1-AP | 1:1000 |
| CD133 Monoclonal antibody | Proteintech | 66666-1-Ig | 1:1000 |
| Anti-Ki67 | Abcam | ab15580 | 1:400 |
| Anti-mouse IgG | ZSGB-BIO | ZB-2305 | 1:5000 |
| anti-rabbit IgG | ZSGB-BIO | ZB-2301 | 1:5000 |
| BMF (E5U2J) Rabbit mAb | CST | #50542 | 1:200 |
| BMF Polyclonal antibody | Proteintech | 18298-1-AP | 1:500 |
| BAX Antibody | CST | #2772 | 1:1000 |
| BCL2 Polyclonal antibody | Proteintech | 12789-1-AP | 1:1000 |
| alpha Tubulin | GeneTex | GTX112141 | 1:1000 |

| Supplementary Table 4. **Information of tumor specimens for PDXs** | | | | | |
| --- | --- | --- | --- | --- | --- |
| Case ID. | Histology | WHO Grage | Gender | | Age |
| GBM1 | High grade glioma | Ⅲ-Ⅳ | Female | | 47 |
| GBM2 | Glioblastoma | Ⅳ | | Male | 29 |
|  | | | | | |

Captions for Data S1

The Δbeta-score and the difference of positive enriched genes in CRISPR/Cas9 screening between LD80 and DMSO treatment group.
